# Bioconversion of Mushroom Chitin-Rich Waste into Valuable Chitin Oligosaccharides Using a Combined Approach of Biocatalysis and Precision Fermentation

**DOI:** 10.1021/acs.jafc.5c00928

**Published:** 2025-04-14

**Authors:** Alex Windels, Luna Declerck, Sofie Snoeck, Wouter Demeester, Chiara Guidi, Tom Desmet, Marjan De Mey

**Affiliations:** Centre for Synthetic Biology, Ghent University, Ghent 9000, Belgium

**Keywords:** mushroom waste, chitin, chitin oligosaccharides, GlcNAc, biocatalysis, fermentation

## Abstract

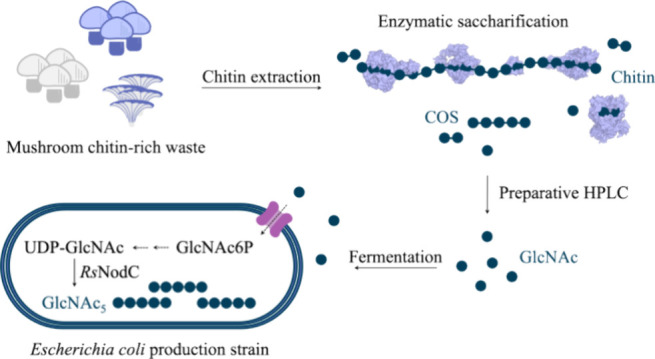

The shift toward a circular economy has increased efforts to derive valuable chemicals from renewable resources, including chitin-rich waste. Mushroom cultivation generates significant waste, particularly the stalks left behind on breeding beds, which contain a substantial amount of chitin with untapped potential. This research establishes a proof of concept for valorizing this waste stream by converting it into valuable chitin oligosaccharides, which have applications across food, feed, agriculture, and pharmaceuticals. Using a combined approach of enzymatic saccharification with five chitinolytic enzymes, followed by precision fermentation of the resulting *N-*acetyl-d-glucosamine (GlcNAc), we successfully produced defined chitinpentaose. Chitin extracted from *Agaricus bisporus* brown demonstrated the highest saccharification efficiency, achieving a GlcNAc conversion of 31 ± 1% (w/w). Our findings highlight the necessity of purifying the saccharification product to ensure product specificity during fermentation, although the production strain’s growth remained suboptimal compared to commercially available GlcNAc. Using an engineered *E. coli* strain, we achieved pure chitinpentaose, with a yield of 0.0327 g/L at a 10 mL scale and production levels (g/OD_600_) comparable to those obtained with HPLC-grade commercial GlcNAc. This study provides a foundation for further research aimed at improving biocatalyst recycling and optimizing the growth phase, thereby enhancing the cost-efficiency and scalability of this sustainable bioconversion process.

## 1 Introduction

Approximately 200 years ago, in 1811, French chemist Henri Braconnot was the first to extract a highly insoluble substance from fungi, naming it fongine. Later renamed chitin by Auguste Odier in 1823, this highly crystalline polymer composed of β-1,4 linked *N*-acetyl-d-glucosamine (GlcNAc) units, has since been extensively studied for its structural role in fungi, insects, crustaceans and mollusks.^1−4^ Chitin and its derivatives, including chitin oligosaccharides (*N-*acetyl-d-glucosamine oligomers; COS) and their (partially) deacetylated analogues chitosan and chitosan oligosaccharides (*N-*acetyl-d-glucosamine and d-glucosamine oligomers; paCOS), hereafter all referred to as COS, have garnered significant interest due to their diverse biological and biomedical properties. These include anti-inflammatory, antioxidant, antimicrobial, immune-stimulating, and anticancer activities, making them valuable for medical, pharmaceutical, food, and agricultural applications.^5−10^ For example, chitinbiose (GlcNAc_2_) is reported to possess food-preservative qualities, while both chitopentaose (GlcN_5_) and chitohexaose (GlcN_6_) enhance plant stress resistance.^11,12^ These biological activities depend on factors such as degree of polymerization (DP), degree of acetylation (DA), and pattern of acetylation (PA), underscoring their immense potential. For instance, the various combinations of DA and PA in chitinpentaose (GlcNAc_5_) can theoretically yield up to 40 distinct molecules, highlighting the versatility of these oligomers.

The push toward a circular economy has intensified the focus on deriving valuable chemicals from renewable resources, such as chitin waste. The seafood industry generates an estimated 6–8 million tons of crustacean shell waste annually, with 20–30% of this waste comprising chitin, depending on species and seasonal factors.^13,14^ Insects represent another significant chitin source, offering advantages over crustaceans due to their nonseasonal availability, rapid reproduction, and the global expansion of insect farming.^15^ Chitin-rich byproducts from insect farming provide a sustainable chitin resource, with chitin content ranging from 5 to 35%.^16^ However, chitin from animal sources is less favored in the food and feed industry due to potential allergenic reactions, leading to increased interest in chitin derived from fungi. Annually, 40 million tons of mushrooms are produced globally, with over 1 million tons grown in Europe. Popular species such as *Agaricus bisporus* (button mushroom), *Lentinula edodes* (shiitake), and *Pleurotus* spp. (oyster mushrooms) produce approximately 5 kg of waste for every one kg grown.^17−19^ This waste consists of spent mushroom substrates, residual fungal mycelium and nonmarketable mushroom parts.^20^ Rich in protein, vitamins, and nutraceuticals, it also contains significant amounts of chitin. For instance, *A. bisporus* has a chitin content of up to 10% dry weight, with a DA ranging between 75.8 and 87.6% and no notable differences between pileus and stems.^21,22^ Additionally, approximately 15% of cultivated mushrooms consist of stems that do not reach consumers, creating a substantial waste stream that holds potential for valorization.

Mushroom-derived chitin is a promising resource for producing COS. One method for depolymerizing chitin is chemical hydrolysis, which employs strong acids like hydrochloric acid. The process involves selective precipitation, ultrafiltration, and spray drying to obtain oligosaccharides with specific molecular weights.^23^ Alternatively, oxidative depolymerization can be achieved using hydrogen peroxide or NaNO_2_.^24,25^ While these chemical methods are well established, they have drawbacks, such as producing heterogeneous degradation products that require extensive purification, and the presence of residual solvents and tributylamine, which impart a bitter taste, limiting their applications in the food industry.^26^

Enzymatic hydrolysis provides a more sustainable alternative, utilizing chitinases (EC 3.2.1.14), chitin deacetylases (EC 3.5.1.41) and chitosanases (EC 3.2.1.132) to break down chitin under mild conditions without undesired side products. While advantageous, the resulting heterogeneity in COS composition remains challenging.^27,28^ An alternative method for obtaining high-value molecules such as COS involves industrial biotechnology, more specifically microbial cell factories. A variety of microorganisms produce chitin, chitosan or COS-derivatives.^29,30^ However, native background mechanisms and pathways influence final product specificity.^31^ Therefore, this and other studies put forward non-native producers as ideal candidates for defined COS-production.^32,33^ By leveraging metabolic engineering, microbial hosts can be tailored to efficiently synthesize non-native compounds. For example, by rewiring the hexosamine biosynthesis pathway and tapping into the native UDP-GlcNAc pool of hosts such as *Escherichia coli* (*E. coli*), it is possible to heterologously produce various mono-, oligo-, and polysaccharides, including human milk oligosaccharides and hyaluronic acid.^34^ However, because metabolic processes are tightly regulated, modifying these pathways can have significant consequences for the microorganism, leading to decreased growth, impaired protein synthesis, and other adverse effects.^35^

**Figure 1:**
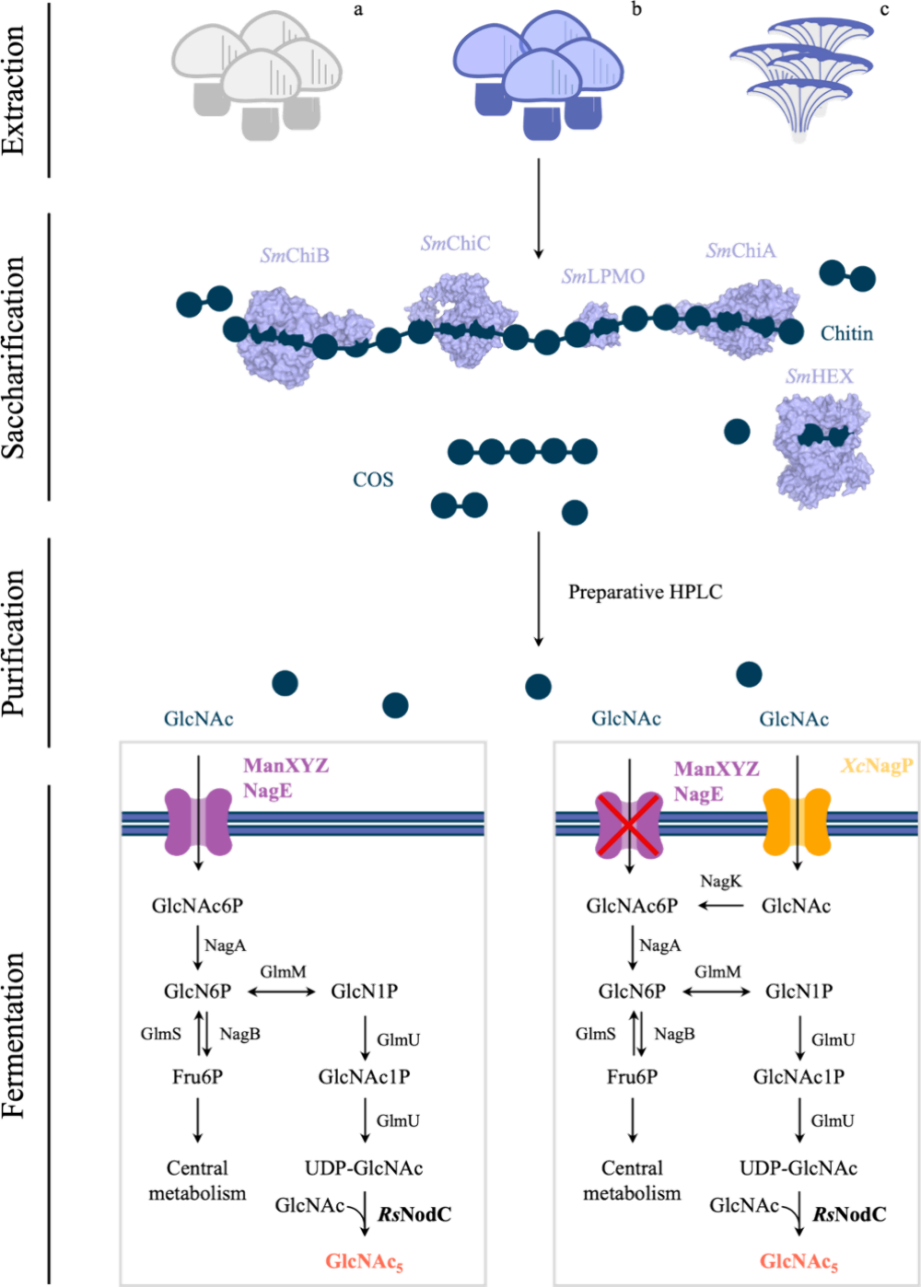
Schematic overview of the combined enzymatic saccharification and precision fermentation approach producing chitinpentaose (GlcNAc_5_) starting from mushroom-waste chitin derived from *Agaricus bisporus* (*A. bisporus*) white (a), *A. bisporus* brown (b) or *Pleurotus ostreatus* (c). The process includes four main steps: (i) extraction of chitin from mushroom stems, (ii) enzymatic saccharification of chitin using five chitinolytic enzymes from *Serratia marcescens*: Chitinase A (*Sm*ChiA), Chitinase B (*Sm*ChiB), Chitinase C (*Sm*ChiC), lytic polysaccharide monooxygenase LPMO10A (*Sm*LPMO) and β-*N*-acetylhexosaminidase (*Sm*HEX), (iii) purification of *N*-acetyl-d-glucosamine (GlcNAc) using preparative liquid chromatography and (iv) precision fermentation with GlcNAc as the carbon source. The *Escherichia coli* 4 knockout strain (Table 1) used in this process has the native manXYZ PTS and NagE transporter systems knocked out and is engineered to express an alternative non-PTS dependent permease NagP from *Xanthomonas campestris* (*Xc*NagP). Heterologous expression of chitin oligosaccharide synthase NodC from *Rhizobium* species GRH2 (*Rs*NodC) enables in the production of GlcNAc_5_. GlcNAc = *N*-acetylglucosamine (solid blue circle), GlcN = glucosamine, COS = chitin oligosaccharides, HPLC = High Performance Liquid Chromatography, P = phosphate, Fru = fructose, *Xc* = *Xanthomonas campestris*, *Rs* = *Rhizobium* species GRH2, *Sm* = *Serratia marcescens*.

Biomanufacturing processes still often use glucose as a primary carbon source due to its availability and efficient uptake, favoring unit economics and eventual product costs. However, challenges persist in making *E. coli*-based fermentation both cost-effective and sustainable for defined COS production, starting from glucose. Therefore, transitioning to waste-derived carbon sources like GlcNAc could address these challenges while meeting the production demands for agrifood applications and supporting the EU Green Deal’s circular bioeconomy goals. GlcNAc, a fundamental building block of COS, serves as an ideal substrate for direct COS biosynthesis in *E. coli*. To optimize its utilization, the growth and production phases can be decoupled by employing an alternative carbon source for cell growth while reserving GlcNAc exclusively for COS synthesis. To this end, GlcNAc can be imported via the non-PTS-dependent permease NagP from *Xanthomonas campestris* (*Xc*NagP, Figure 1), thereby circumventing carbon catabolite repression. This mechanism facilitates the decoupling of growth and COS production phases, ultimately enhancing the sustainability and efficiency of the production process (Figure 1).^36^

In this study, we integrated two complementary approaches: (i) to reduce product heterogeneity from enzymatic degradation, the substrate was saccharified into its monosaccharide component GlcNAc using β-*N*-acetylhexosaminidases (EC 3.2.1.52). These enzymes cleave GlcNAc residues from the nonreducing ends of polymers and oligosaccharides, producing a more uniform product. (ii) This GlcNAc, derived from a mushroom (*A. bisporus*) chitin waste stream, was then used as a sustainable carbon source for precision fermentation of COS. Specifically, we combined enzymatic saccharification of chitin using five chitinolytic enzymes from *Serratia marcescens* with the heterologous expression of chitin oligosaccharide synthase from *Rhizobium* species GRH2 (*Rs*NodC) in *E. coli* to produce chitinpentaose (GlcNAc_5_). This strategy ensures the sustainable production of high-quality, well-defined COS while effectively valorizing mushroom waste streams (Figure 1).

## 2 Materials and Methods

All chemicals were purchased from Merck, unless noted otherwise.

### 2.1 Strains, Plasmids, and Culture Conditions

#### 2.1.1 Media and Culture Conditions

Lysogeny broth (LB) was used to grow the strains for routine cloning, enzyme production experiments and precultures for both microtiter plate (MTP) and shake flask experiments (10 g/L tryptone, 5 g/L NaCl, and 5 g/L yeast extract). Liquid cultures were grown at 30 °C and shaken at 200 rpm (LS-X (5 cm orbit), Adolf Kühner AG, Switzerland). Minimal medium (MM) was used for MTP and shake flask experiments. MM was composed of 2 g/L NH_4_Cl, 5 g/L (NH_4_)_2_SO_4_, 3 g/L KH_2_PO_4_, 7.3 g/L K_2_HPO_4_, 8.4 g/L MOPS, 0.5 g/L NaCl, 0.5 g/L MgSO_4_·7H_2_O, a carbon source (2.8 g/L GlcNAc, 6.9 g/L GlcNAc, 13.81 g/L GlcNAc, or 16.5 g/L glucose), 1 mL/L trace element solution and 100 μL/L molybdate solution. Trace element solution consisted of 3.6 g/L FeCl_2_·4H_2_O, 5 g/L CaCl_2_·2H_2_O, 1.3 g/L MnCl_2_·2H_2_O, 0.38 g/L CuCl_2_·2H_2_O, 0.5 g/L CoCl_2_·6H_2_O, 0.94 g/L ZnCl_2_, 0.0311 g/L H_3_BO_4_, 0.4 g/L Na_2_EDTA·2H_2_O and 1.01 g/L thiamine·HCl. The molybdate solution contained 0.967 g/L Na_2_MoO_4_·2H_2_O. Carbon sources (glucose, High-performance liquid chromatography (HPLC)-grade GlcNAc (Chem-Lab NV, Belgium) or mushroom-derived GlcNAc) were added to achieve equimolar amounts of carbon between the different sources where relevant. To avoid Maillard reaction during autoclaving, the carbon source and magnesium sulfate were separately filter sterilized (250 mL filter unit, 0.2 μm PES membrane; VWR, Belgium). The other salts and components were set to a pH of 7 using KOH before being autoclaved (121 °C, 30 min, 1.02 atm). The two solutions were mixed and supplemented with the filter sterilized (Sterile syringe filter 0.2 μm PES; VWR) vitamin mix and molybdate solution. If required, the culture medium was supplemented with appropriate antibiotics. Stock concentrations for antibiotics were 100 mg/mL for ampicillin, 50 mg/mL for kanamycin and 25 mg/mL for chloramphenicol. Antibiotic stocks were filter sterilized and diluted 1000× for cell culture experiments.

For the experiment testing the toxicity of ascorbic acid, GlcNAc was kept separate from the rest of the MM. Different concentrations of ascorbic acid were added to the GlcNAc, to account for a final concentration in the MM of 0.176, 0.455, or 0.910 g/L ascorbic acid. To test the influence of heating the ascorbic acid, the GlcNAc and ascorbic acid were heated for 10 min at 100 °C, before being filter sterilized (Sterile syringe filter 0.2 μm PES; VWR) and added to the rest of the MM.

All polymerase chain reactions (PCR) were done using either PrimeStar HS (Takara, Westburg, The Netherlands) or PrimeStar Gxl DNA polymerase (Takara). The resulting DNA fragments were purified using the innuPREP PCRpure Kit (Analytic Jena AG, Germany).

#### 2.1.2 Gene Synthesis and Cloning

All coding sequences are listed in Table S1. The sequences of *Sm*ChiA (UniProt: P07254), *Sm*ChiB (UniProt: P11797), *Sm*ChiC (UniProt: X2KWS3), *Sm*LPMO (UniProt: P9WEM3) and *Sm*HEX (UniProt: Q54468) were codon-optimized for expression in *E. coli*, synthesized and subcloned into the *Nhe*I and *XHo*I restriction sites of a pET21a vector by Life Technologies (Belgium). All genes contained a C-terminal His_6_-tag. The plasmids were used for transformation to electrocompetent *E. coli* BL21(DE3) (Invitrogen, USA) cells.

#### 2.1.3 Production Strains

Bacterial strains used for precision fermentation are shown in Table 1. For the construction and storage of plasmids, One Shot Top10 Chemically Competent *E. coli* (Invitrogen, USA) was used. Knockouts of *chb*BCARFG, *chi*A, *nag*Z, nag*E*, and *man*XYZ were made using the method developed by Datsenko and Wanner in *E. coli* K12 MG1655 Δ*chb*BCARFGΔ*chi*AΔ*nag*Z (3KO) and *E. coli* K12 MG1655 (mutant version of ATCC 47076, additional deletions resulting in Δ*yna*J Δ*usp*E Δ*fnr* Δ*cct* Δ*abg*T Δ*abg*B Δ*abg*A Δ*abg*R Δ*smr*A Δ*yda*M Δ*yda*N Δ*dbp*A Δ*ttc*A Δ*int*R Δ*rec*T Δ*rec*E). For the experiments, *E. coli* K12 MG1655 Δ*chb*BCARFGΔ*chi*AΔ*nag*Z (3KO) and *E. coli* K12 MG1655 Δ*chb*BCARFGΔ*chi*AΔ*nag*EΔ*man*XYZ (4KO) were transformed with the corresponding plasmids (Table 1) via electroporation or heat shock.^37,38^

**Table 1:** *Escherichia coli* Strains Used for the Precision Fermentation Experiments^a^

strain	genetic background	plasmid details: p[ori][AB][Prom-RBS-CDS-Term]_*n*_
WT	*E. coli* K12 MG1655	
3KO	*E. coli* K12 MG1655 Δ*chb*BCARFGΔ*chi*AΔ*nag*Z	
3KO_A5	*E. coli* K12 MG1655 Δ*chb*BCARFGΔ*chi*AΔ*nag*Z	p[BR322][Amp][P_14_-RBS(T7)-*Rsnod*C-T(TT7)]
3KO_PaA5	*E. coli* K12 MG1655 Δ*chb*BCARFGΔ*chi*AΔ*nag*Z	p[BR322][Kan][P_14_-RBS(T7)-*Rsnod*C-T(TT5-T7term)][P_22_-RBS5- *Rsnod*B-T(TT7-M13)]
4KO_A5_GlcNAc_1	*E. coli* K12 MG1655 Δ*chb*BCARFGΔ*chi*AΔ*nag*EΔ*man*XYZ	p[BR322][Kan][P_nagP2_-RBS(P_nagP2_)-*Xcnag*P-T(TT3-rrnD1-T1)] + P[15A][Chl][P_14_-RBS(T7)-*Rsnod*C-T(TT5-T7term)][nonCDS]
4KO_A5_GlcNAc_2	*E. coli* K12 MG1655 Δ*chb*BCARFGΔ*chi*AΔ*nag*EΔ*man*XYZ	p[BR322][Kan][P_nagP3_-RBS(P_nagP3_)-*Xcnag*P-T(TT3-rrnD1-T1)] + P[15A][Chl][P_14_-RBS(T7)-*Rsnod*C-T(TT5-T7term)][nonCDS]

a *E. coli* = *Escherichia coli*, WT = wild-type, KO = knockout, ori = origin of replication, AB = antibiotic resistance, Prom = promoter, RBS = ribosome binding site, CDS = coding DNA sequence, Term = terminator, Kan = kanamycin, Chlo = chloramphenicol, Amp = ampicillin, A5 = fully acetylated chitinpentaose, PaA5 = partially acetylated chitinpentaose, GlcNAc = *N*-acetylglucosamine

#### 2.1.4 Plasmid Construction

An overview of the plasmids used is listed in Table 1. The plasmids containing pBR322 origin of replication (ori) are medium-copy vectors (pET22b) with an ampicillin or kanamycin resistance marker (full sequence of the backbones can be found in Table S2).^39^ They were constructed with Circular Polymerase Extension Cloning (CPEC), using Q5 High-Fidelity DNA polymerase (Bioké, The Netherlands).^40^ The plasmid containing a medium copy p15A ori and chloramphenicol antibiotics resistance gene (full sequence of the backbone can be found in Table S2) was assembled using an in-house assembly system based on the Standard European Vector Architecture (SEVA) plasmid toolkit.^41^ The promoter (P_14_), 5′ untranslated region (5′UTR) (T7 5′UTR), genes (*Rs*NodC, nonCDS) and terminator (TT5-T7term) were stored in Level0 plasmids. The two transcription units (P_14_-RBS(T7) *Rs*NodC_T(TT5-T7term) and nonCDS) were assembled into Level1 plasmids and finally combined in a Level2 plasmid. This in house-assembly system uses Golden Gate (GG) assembly, which uses Type II restriction enzymes and enables directional, scar free cloning.^42^ Level0 and Level2 assembly require BsmBI, Level1 uses BsaI (Bioké, Leiden, The Netherlands). Level0 parts were derived from in-house plasmids or were ordered at Integrated DNA Technologies BVBA (Belgium) or GeneArt (Thermo Fisher Scientific, Belgium) and assembled into Level0 acceptor plasmids using either CPEC or GG. The promoter and ribosome binding site sequences of *Xcnag*P_1_ and *Xcnag*P_2_ were selected from a promoter/RBS library derived from the P_22_ promoter and a synthetic 5′ UTR, in which the spacer region was fully randomized and the RBS semirandomized (MHGVMGGATS) (Table S3). The transcription rate (TR) and translation initiation rate (TIR) were determined using the Salis Calculator (Table S3).^43,44^ These estimations indicated that *Xcnag*P_1_ resulted in the highest amount of the *Xc*NagP permease. The DNA sequence of *Xcnag*P (codon optimized), the promoter, 5′UTR and terminator sequences can be found in Table S1.

One Shot Top10 (Invitrogen) chemically competent *E. coli* was transformed with the plasmids and their presence in the colonies was verified by colony PCR. The DNA sequences of the plasmids were verified by growing positive colonies overnight, prepping the plasmids using QIAprep Spin Miniprep Kit (Qiagen, The Netherlands) and sending them for Sanger sequencing to Macrogen (Macrogen Inc., The Netherlands). Finished plasmids were then transformed into the desired background strain.

#### 2.1.5 Adaptive Evolution

The strains of 4KO_A5_GlcNAc_1 and 4KO_A5_GlcNAc_2 were incubated in minimal medium with GlcNAc (MMGlcNAc), containing 50 mg/mL kanamycin and 25 mg/mL chloramphenicol, over multiple passages to promote adaptive evolution. Initially, a 5 mL LB preculture was grown overnight at 30 °C with shaking at 200 rpm. The next day, 5 mL of this preculture was subcultured into 50 mL of MMGlcNAc containing 13.81 g/L GlcNAc. Cultures were grown at 30 °C with shaking at 200 rpm until reaching stationary phase, after which a 1% inoculum was immediately subcultured into fresh 50 mL MMGlcNAc for continued adaptation. The evolved strains were stored in cryovials at −80 °C.

### 2.2 Production of Chitinpentaose

#### 2.2.1 Chitin Extraction

Stems from *Agaricus bisporus* white, *Agaricus bisporus* brown, and *Pleurotus ostreatus* were provided by Inagro (Belgium). Upon arrival, the stems were cleaned and stored at −20 °C. They were then freeze-dried using a Lyovapor L-200 freeze-dryer (Buchi, USA). Chitin extraction was performed according to Synowiecki and Al-Khateeb with the following modifications.^45^ For deproteinization, 4 g of the dry product was mixed with 120 mL of 1 M NaOH and heated at 80 °C for 2 h. After another freeze-drying step, the stems were washed and centrifuged iteratively until a neutral pH was achieved. Subsequently, the samples were treated with 100 mL of 2% (v/v) acetic acid per gram of mushroom at 95 °C for 6 h. Following this treatment, the samples were washed and centrifuged repeatedly until neutral pH was reached. The resulting chitin was then milled using a Fritsch Pulverisette (Belgium) 14 equipped with a 200 μm filter.

#### 2.2.2 Protein Expression and Purification

For recombinant protein expression of the chitinolytic enzymes, an overnight culture (2% inoculum) of *E. coli* BL21(DE3) cells carrying the desired pET21a expression plasmid was used to inoculate 250 mL of LB medium supplemented with 50 μg/mL ampicillin. The culture was incubated at 37 °C with shaking at 200 rpm until the optical density at 600 nm (OD600) reached 0.6. The temperature was then lowered to 18 °C for 2 h to acclimate the cells. Protein expression was induced by adding isopropyl β-d-1-thiogalactopyranoside (IPTG) to a final concentration of 0.5 mM, and the culture was incubated at 18 °C for an additional 16 h. Following incubation, the cells were harvested by centrifugation, and the resulting cell pellets were frozen at −20 °C for a minimum of 4 h.

To extract and purify the enzymes, the cell pellets were thawed and resuspended in 8 mL of lysis buffer (10 mM imidazole, 0.1 mM phenylmethylsulfonyl fluoride (PMSF), 1 mg/mL lysozyme from chicken egg white, and 50 mM phosphate-buffered saline (PBS); pH 7.4). The suspension was incubated on ice for 30 min. Following incubation, the lysate was sonicated three times for 3 min each (using a Branson Sonifier 450, level 3, 50% duty cycle). The cell extract was then separated from the cell debris by centrifugation at 20,000*g* for 1 h at 4 °C. The supernatant was purified using nickel-nitrilotriacetic acid (Ni-NTA) chromatography according to the manufacturer’s instructions (HisPur Ni-NTA; Thermo Fisher Scientific, Belgium). The purified enzyme fraction was concentrated, and the buffer was exchanged to 50 mM 2-morpholinoethanesulfonic acid (MES) at pH 6.5 using an Amicon centrifugal filter unit with a 30 kDa cutoff. The extinction coefficient and molecular weight of all enzymes were calculated using the ProtParam tool on the ExPASy server (http://web.expasy.org/protparam). Protein concentrations were measured using a NanoDrop ND-1000 spectrophotometer at 280 nm (Thermo Fisher Scientific, Belgium). Purified enzyme preparations were stored at −20 °C.

#### 2.2.3 Enzymatic Saccharification of Mushroom-Derived Chitin

Chitin hydrolysis was performed following the method described by Mekasha and coauthors with the following modifications.^46^ Reactions were conducted in 0.5 mL of 50 mM MES buffer (pH 6.5) containing 1 mM ascorbic acid, 15 mg/mL chitin, and 15 mg enzyme mixture, as described in Table 2, per gram of chitin. The reactions were incubated at 42 °C with shaking at 800 rpm in an Eppendorf Comfort ThermoMixer. For HPAEC-PAD analysis, 20 μL reaction samples were added to 20 μL of 50 mM sulfuric acid and incubated at 100 °C for 10 min. The samples were then diluted and filtered using 0.22 μm syringe filters (Phenomenex, The Netherlands).

**Table 2:** *Serratia marcescens* (*Sm*) Enzymes for the Saccharification of Chitin^a^

enzyme	abbreviation	enzyme dosage (% w/w)
*Serratia marcescens* chitinase A	*Sm*ChiA	40
*Serratia marcescens* chitinase B	*Sm*ChiB	30
*Serratia marcescens* chitinase C	*Sm*ChiC	15
*Serratia marcescens* LPMO10A	*Sm*LPMO	3
*Serratia marcescens* β-*N*-acetylhexosaminidase	*Sm*HEX	12

a The table shows the ratio of every enzyme in the cocktail of which the total enzyme dosage was 15 mg enzyme/g chitin is shown. W = weight.

GlcNAc was purified from the reaction mixture using a preparative HPLC system. The AZURA Sugar Purification System (Knauer, Germany) was employed, equipped with a 75 °C heated Vertex Plus AX column (250 mm × 30 mm) loaded with Knauer Eurokat Na-resin (mesh size 25–56 μm) and a refractive index detector (RID). Elution was carried out using ultrapure water as the solvent at 80 °C. Initially, the column was flushed with ultrapure water at a flow rate of 7.5 mL/min for 1 min. Subsequently, 1.5 mL of the sample was injected onto the column at a flow rate of 1.5 mL/min. Following injection, ultrapure water was used as the eluent to elute the carbohydrates at a flow rate of 7.5 mL/min. The GlcNAc fraction was collected while other fractions were discarded. After 20 min, a new run was automatically initiated. The collected GlcNAc fraction was frozen in Falcon tubes at–80 °C and then freeze-dried using a Lyovapor L-200 feeze dryer (Buchi, USA).

To assess GlcNAc stability in MM, three samples were tested: 13.8 mg/mL of HPLC-grade GlcNAc, unpurified GlcNAc obtained from enzymatic hydrolysis of *A. bisporus* brown-derived chitin, and prep-LC purified GlcNAc from the same source. Each sample was incubated at 30 °C with shaking at 350 rpm for 48 h.

#### 2.2.4 Precision Fermentation for the Production of Chitinpentaose

To analyze the growth profiles of the strains, a screening was done on MTP scale. Precultures were grown in 150 μL of LB containing the appropriate antibiotics in Transparent CELLSTAR© polystyrene 96-well MTPs (Greiner Bio-One, Belgium) covered with a clear polystyrene lid with condensation rings (Greiner Bio-One, Belgium). Precultures were shaken for 24 h at 30 °C and 800 rpm on the Compact Digital Microplate Shaker (3 mm orbit, Thermo Fisher Scientific, Belgium). Afterward, they were inoculated (1%) in the respective MM containing appropriate antibiotics. To follow up growth, the Tecan M200 Pro (MNano+) plate reader (Tecan, Belgium) was used at 30 °C and 200 rpm by measuring the OD600 every 10 min for 50 h.

To analyze COS production, precultures (triplicate) were grown in LB containing appropriate antibiotics for 24 h at 30 °C and 200 rpm (5 cm orbit, LS-X, Adolf Kühner AG, Switzerland). Next, they were inoculated (1%) in 10 mL MM with appropriate antibiotics (50 mL shake flask). To follow up growth, samples for OD_600_ were regularly taken and measured with the Fisherbrand Cell Density Meter (Thermo Fisher Scientific, Belgium). After 24 and 48 h, COS samples were taken. Therefore, 1.5 mL of culture was transferred to a 1.5 mL tube and centrifuged for 10 min at 20,627*g* (Sigma 1–16, Sigma Laborzentrifugen GmbH, Germany). The pellet was dried and then stored at −80 °C until further use.

### 2.3 HPLC Analysis of COS Products

Hydrolysis products (GlcNAc to GlcNAc_6_) from the enzymatic saccharification reactions and GlcNAc stability tests in MM were analyzed using a Dionex HPAEC-PAD ICS-6000 system (Thermo Fisher Scientific, Belgium) equipped with a CarboPac PA20 150 mm analytical column and a 30 mm guard column. The mobile phase was 30 mM NaOH at a flow rate of 0.5 mL/min at 30 °C for 10 min. COS were quantified using standards obtained from Megazyme.

Cell pellets obtained from the precision fermentation process for the production of chitinpentaose were thawed and dissolved in 150 μL of 60% acetonitrile (AcN) (Chem-Lab NV, Belgium) in milli-Q water (mQ) after which the samples were centrifuged for 10 min at 20,627*g* (Sigma 1-16, Sigma Laborzentrifugen GmbH). Finally, 100 μL of the supernatant was transferred to a vial (VerexTM Vial, 9 mm Screw Top, μVial i3 (Qsert), Clear 33, No Patch; Phenomenex) and closed off with a cap (VerexTM Cap (preassembled), 9 mm, Screw top, w/PTFE/Silicone presplit septa, blue; Phenomenex) for analysis. Details for the HPLC run can be found in Table 3. Analysis was done by hydrophilic interaction chromatography (HILIC), with the Kinetix 2.6 μm HILIC 100A column (2.6 μm, 4.6 mm × 150 mm) (Phenomenex, The Netherlands) and a precolumn consisting of a SecurityGuard ULTRA Cartridges, UHPLC HILIC 4.6 mm ID Columns-AJ0-8772 (Phenomenex, The Netherlands) and a SecurityGuard ULTRA Holder, for UHPLC Columns 2.1 to 4.6 mm ID-AJ0-9000 (Phenomenex, The Netherlands).

**Table 3:** High-Performance Liquid Chromatography (HPLC) Analysis for the Quantification of Chitin Oligosaccharide Production^a^

general characteristics		method		
column	Kinetix 2.6 μm HILIC 100A column (2.6 μm, 4.6 mm × 150 mm)	**time (min)**	**%A**	**%B**
elution type	gradient	0–8	85	15
		8–20	55	45
eluens A	100% AcN + 0.1 v/v % FA	20–27	50	50
		27–30	85	15
		30–40	85	15
eluens B	10 mM NH_4_–FA + 0.1 v/v % FA			
flow	1.0 mL/min			
column T	45 °C	**component**	**elution time (min)**	
method length	40 min	A5	15.25–16.50	
detection	ELSD			
injection volume	2 μL			

a Characteristics of the HPLC and its method. AcN = acetonitrile, FA = formic acid, A5 = fully acetylated chitinpentaose, column T = column temperature, ELSD = evaporative light scattering detector.

### 2.4 Data Analysis

For statistical analysis, a significance level of 0.05 was applied. The Python package scipy.stats was used to perform a two-sample *t* test or a one-way ANOVA, followed by Tukey’s HSD test for posthoc analysis to account for multiple comparisons.

## 3 Results and Discussion

### 3.1 Saccharification of Mushroom-Derived Chitin

The saccharification efficiency of three mushroom-derived chitin sources, extracted from *Agaricus bisporus* white (white button mushroom), *Agaricus bisporus* brown (Portobello), and *Pleurotus ostreatus* (oyster mushroom) - was evaluated using a suite of five chitinolytic enzymes from *Serratia marcescens*: *Sm*ChiA, *Sm*ChiB, *Sm*ChiC, *Sm*LPMO, and *Sm*HEX (Table 2).^47^*Sm*ChiA and *Sm*ChiB are processive exochitinases that degrade the chitin chain from the reducing and nonreducing ends, respectively, producing primarily chitinbiose and GlcNAc.^48^*Sm*ChiC, in contrast, is a nonprocessive endochitinase that randomly cleaves the chitin polymer, resulting in the formation of monosaccharides, disaccharides, and occasionally longer oligosaccharides. Additionally, a lytic polysaccharide monooxygenase (LPMO) is incorporated into the enzyme cocktail, which catalyzes the oxidative cleavage of glycosidic bonds, thereby reducing the recalcitrance of the substrate and making it more amenable to further enzymatic hydrolysis.^46^ To maximize conversion to the monosaccharide level, *Sm*HEX, a β-*N*-acetylhexosaminidase, is included in the mixture, catalyzing the breakdown of oligo- and disaccharides into GlcNAc.^49^

**Figure 2:**
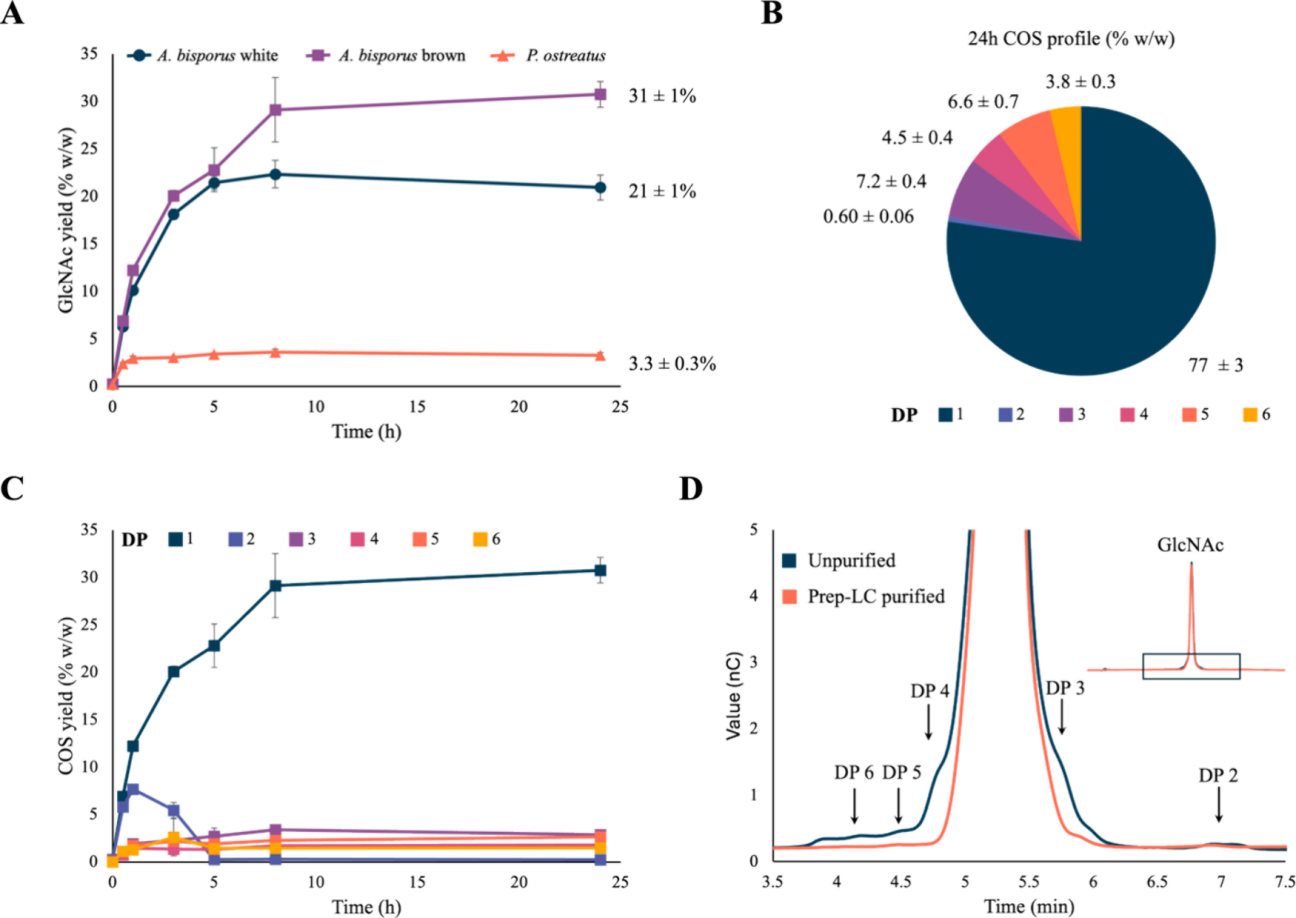
Saccharification of mushroom-waste chitin using five chitinolytic enzymes from *Serratia marcescens*. (A–C) Mean over three biological replicates (*n* = 3) is shown, with error bars depicting the standard error of the mean. (B–D) Reactions performed on *A. bisporus* brown chitin. (A) Comparison of GlcNAc yield among *A. bisporus* white, *A. bisporus* brown and *P. ostreatus* chitin. (B) COS product profile after 24 h enzymatic reaction. (C) COS production during the biocatalytic saccharification process. (D) HPAEC-PAD analysis of unpurified and preparative HPLC-purified saccharification product. DP indicates the degree of polymerization of the chitin oligosaccharides analyzed. COS = chitin oligosaccharides, DP = degree of polymerization, 1–6 = DP of analyzed COS molecules, GlcNAc = *N*-acetylglucosamine, *A. bisporus* = *Agaricus bisporus*, *P. ostreatus* = *Pleurotus ostreatus*, HPAEC-PAD = high performance anion exchange chromatograpy–pulsed amperometric detection, HPLC = high performance liquid chromatography.

The highest yield of GlcNAc, defined as the percentage (w/w) of GlcNAc obtained relative to the initial amount of chitin used as substrate, was obtained from chitin derived from brown *A. bisporus*, resulting in a GlcNAc yield of 31 ± 1% w/w (Figure 2A). After 24 h of reaction, the total chitin conversion to COS was 40 ± 2% w/w with primary products being GlcNAc (77% w/w), chitintriose (7.2% w/w), and chitinpentaose (6.6% w/w) (Figure 2C, Table S4). Chitinbiose is produced in the initial stages of the reaction but is gradually hydrolyzed to GlcNAc by *Sm*HEX, resulting in a final concentration of less than 1% w/w in the product (Figure 2B). In comparison, the total conversion of white *A. bisporus* chitin to COS was lower, at 28 ± 3% w/w, though it exhibited a similar product profile (Table S4 and Figure S1A,B). For *P. ostreatus* chitin, only 6.1 ± 0.8% w/w was converted to COS. The product was predominantly GlcNAc (55%), with higher proportions of chitintriose, chitinpentaose, and chitinhexaose compared to *A. bisporus* chitin, at 18% w/w, 11% w/w, and 10% w/w, respectively (Figure S1C,D). Due to the superior conversion yield, higher efficiency, and relative purity of brown *A. bisporus* chitin, which facilitates downstream purification and improves the final product quality, this chitin source was selected for subsequent reactions.

Although no significant differences in carbohydrate content are expected between the white and brown mushroom chitin, differences in harvesting time and storage conditions may lead to compositional variations, influencing both chitin extraction and saccharification yield (Figure S2).^22,50^ The carbohydrate content between *A. bisporus* and *P. ostreatus* mushrooms is similar; however, *A. bisporus* mushrooms have a higher chitin level, and the extraction protocol used was specifically optimized for this species, which may explain the difference in observed GlcNAc and COS yield.^22,51^ Additionally, *A. bisporus* chitin has a lower crystalline index (63%) compared to *P. ostreatus* chitin (73%), facilitating more efficient hydrolysis.^47,52^ Differences in the degree of *N*-acetylation—66% for *A. bisporus* chitin and 57% for *P. ostreatus* chitin—further contribute to variations in the product profile and saccharification yield.^53^ These factors significantly impact the efficiency and scalability of the conversion process, necessitating optimization of both chitin extraction and saccharification for each specific resource.

Despite the satisfactory yields, the enzyme dosage of 15 mg enzyme per gram of chitin remains prohibitively high for industrial applications, requiring at least a 100-fold reduction to improve economic feasibility.^54^ To enhance the cost-efficiency of the saccharification process and optimize biocatalyst utilization, future research should focus on enzyme recycling strategies, such as immobilization techniques, optimizing reaction conditions and exploring alternative Chitinase homologues.^55^

### 3.2 Production of Chitinpentaose Using Precision Fermentation, Starting from Mushroom-Derived GlcNAc

**Figure 3:**
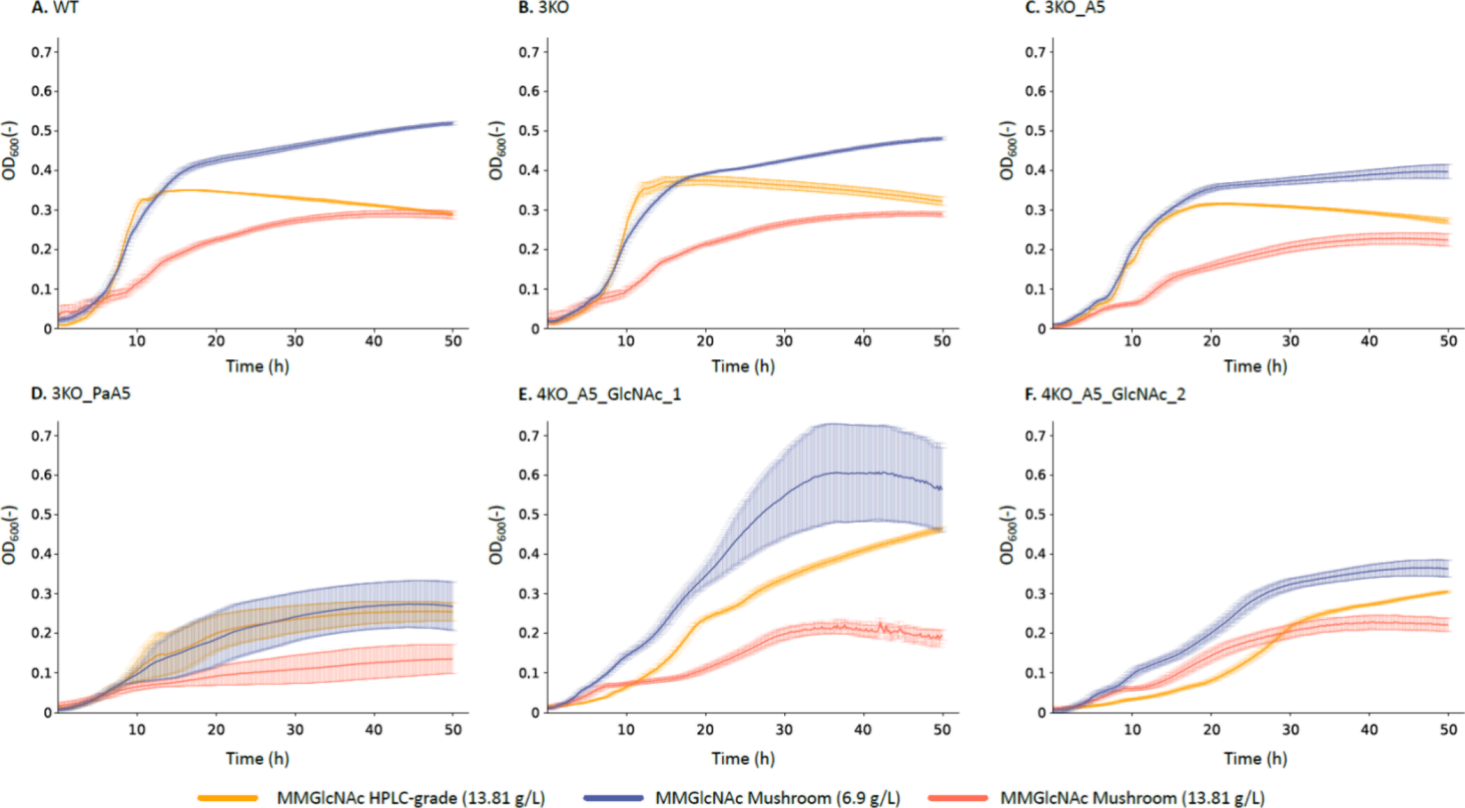
*Escherichia coli* (*E. coli*) growth curves (optical density at 600 nm followed in time) for two mushroom-based GlcNAc (13.81 and 6.9 g/L) minimal media compared to 13.81 g/L commercial, HPLC-grade, shrimp-derived GlcNAc (= HPLC-grade GlcNAc) minimal medium. The mean over four biological replicates (*n* = 4) is shown, with error bars depicting standard error of the mean. Six *E. coli* strains (Table 1) were tested: (A) wild-type MG1655, (B) 3KO, (C) 3KO_A5, (D) 3KO_PaA5, (E) 4KO_A5_GlcNAc_1, (F) 4KO_A5_GlcNAc_2. KO = Knockout, WT = Wild-type, MM = Minimal medium, GlcNAc = *N*-acetylglucosamine, A5 = fully acetylated chitinpentaose, PaA5 = partially acetylated chitinpentaose, OD_600_ = optical density at 600 nm, HPLC = high-performance liquid chromatography.

The unpurified batch of GlcNAc obtained from the brown *A. bisporus* was used as carbon source in minimal medium to produce COS via microbial fermentation. The growth and/or COS production of six *E. coli* strains (Table 1) were tested, using minimal media containing either (i) 13.81 g/L or (ii) 6.9 g/L mushroom-derived GlcNAc as carbon source (Figure 3). These concentrations were selected because they provide the same total carbon input as 16.5 and 8.25 g/L glucose, respectively.^56,57^ These six strains include two controls (wild-type (WT) and 3KO) and four COS producing strains (3KO_A5, 3KO_PaA5, 4KO_A5_GlcNAc_1, and 4KO_A5_GlcNAc_2). In all strains, except the WT, three genes that counteract COS-interfering background mechanisms were knocked out (Table 1).^58^ Strains consisting of the *Rs*NodC enzyme mainly produce the COS molecule chitinpentaose, which is referred to as acetylated 5 (A5), while the combined expression of *Rs*NodC and *Rs*NodB results in a COS mixture composed of fully and partially acetylated chitinpentaose, referred to as partially acetylated 5 (PaA5).^56^ Strains 4KO_A5_GlcNAc_1 and 4KO_A5_GlcNAc_2 lack the native phosphotransferase system (PTSs) for GlcNAc import, but express a GlcNAc permease from *Xanthomonas campestris* (*Xc*NagP).^36^ The difference between these two strains are the promoter and ribosome binding site (RBS) used for the expression of *Xc*NagP, with strain 4KO_A5_GlcNAc_1 containing a stronger promoter and RBS to produce *Xc*NagP (Table S3). This permease does not phosphorylate incoming GlcNAc, avoiding the immediate production of GlcNAc-6P upon uptake (Figure 1).^36^ Using a non-PTS GlcNAc importer would allow simultaneous uptake of GlcNAc and other carbon sources without inducing carbon catabolite repression, a first step in separating COS production from growth.^59^ Moreover, these strains were subjected to an overnight adaptive evolution (AE) experiment which improved growth and total COS production titers (Figures S3 and S4). All strains were tested for COS production on commercial, HPLC-grade, shrimp-derived GlcNAc, from now on referred to as HPLC-grade GlcNAc.

*E. coli* growth was followed in time and growth on unpurified mushroom-derived GlcNAc minimal medium was compared to growth on HPLC-grade GlcNAc minimal medium (Figure 3). All strains showed an overall decrease in specific growth when 13.81 g/L mushroom-derived GlcNAc was added as carbon source, while an improved growth profile was obtained when only 6.9 g/L of mushroom-derived GlcNAc was used. Since growth and final OD were restored at this lower concentration, this suggests that impurities—such as Maillard reaction products, pigments, or other residual compounds—rather than the GlcNAc concentration itself, negatively impact growth.^60−63^ A detailed compositional analysis of the saccharification product is needed to confirm this hypothesis. These findings underscore the importance of further purification steps to enhance the suitability of mushroom-derived GlcNAc as a carbon source.

**Figure 4:**
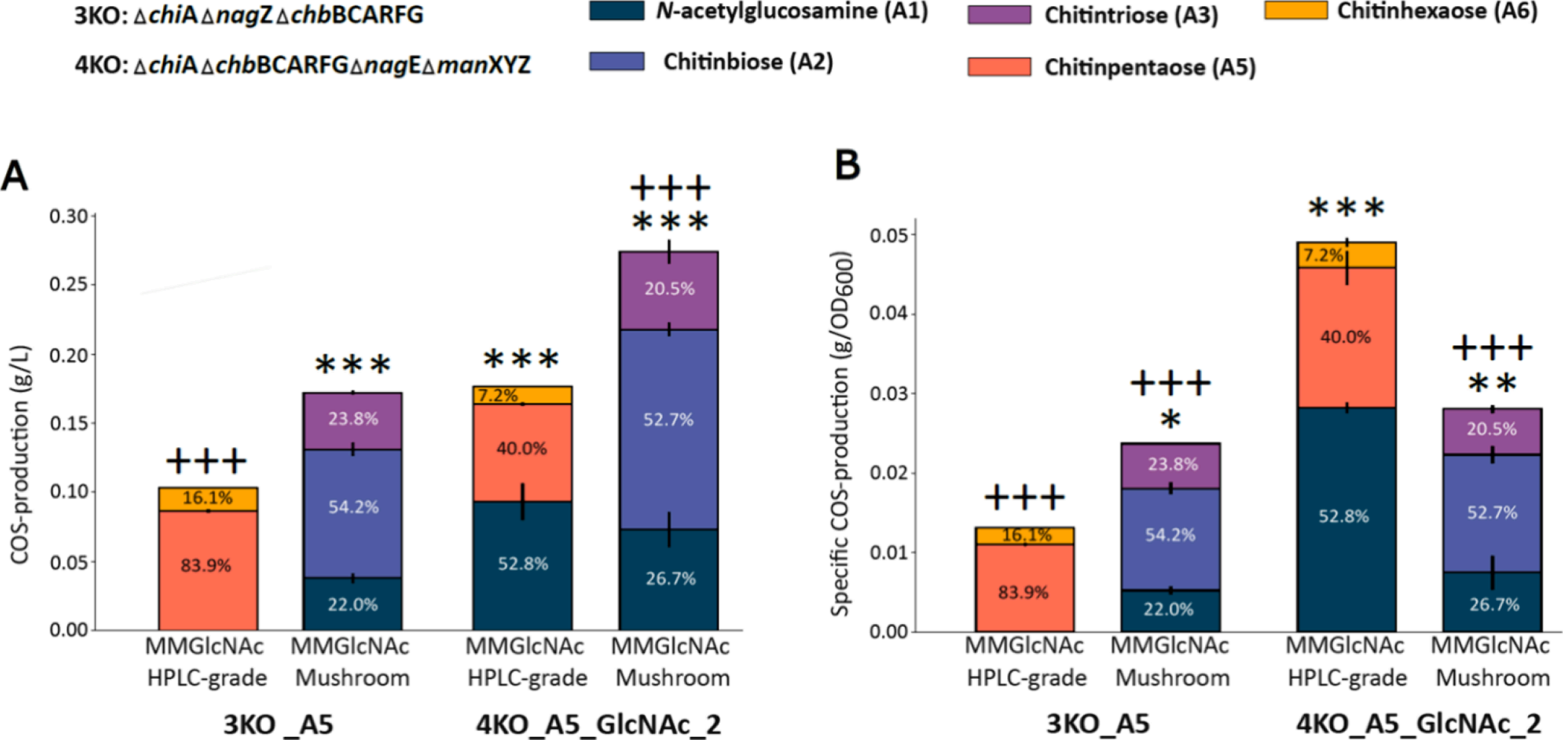
COS production of two *Escherichia coli* strains: 3KO_A5 and 4KO_A5_GlcNAc_2 (see Table 1 for more details on the strains) A mushroom-derived GlcNAc concentration of 13.81 g/L was used. Results were compared to production on 13.81 g/L commercial, HPLC-grade, shrimp-derived GlcNAc (= HPLC-grade GlcNAc) minimal medium. (A) COS production titers and COS production profiles. (B) Specific COS production and COS production profiles. The mean over three biological replicates (*n* = 3) is shown. The error bars represent standard error on the mean. For statistical analysis, one-way ANOVA was performed, using Tukey HSD to adjust for multiple comparison. */+: *p* < 0.05, **/++: *p* < 0.01, ***/+++: *p* < 0.001. The p-values displayed on the figure as * correspond to the comparison with 3KO_A5 grown on HPLC-grade GlcNAc MM, and those displayed as + correspond to the comparison with 4KO_A5_GlcNAc_2 grown on HPLC-grade GlcNAc MM. More details on the statistical tests can be found in Table S5, and of the COS production titers in Table S6. MM = minimal medium, COS = chitin oligosaccharides, GlcNAc = *N*-acetylglucosamine, A5 = fully acetylated chitinpentaose, KO = knockout, HPLC = high-performance liquid chromatography.

The COS production (Figure 4A) and specific production (Figure 4B) titers of the 3KO_A5 and 4KO_A5_GlcNAc_2 strains were tested using 13.81 g/L HPLC-grade and mushroom-derived GlcNAc. These strains were selected for their specificity in chitinpentaose production and in order to make a comparison between COS production when GlcNAc is imported via a PTS versus non-PTS system. From the non-PTS GlcNAc importer strains, 4KO_A5_GlcNAc_2 was chosen since this has the weakest promoter, decreasing metabolic stress levels (Table S3). As expected, intracellular GlcNAc was detected in the 4KO_A5_GlcNAc_2 strain, which was engineered for non-PTS GlcNAc uptake (Figure 1). However, intracellular GlcNAc was also observed in the 3KO_A5 strain when grown on mushroom-derived GlcNAc. Upon import into the cell, GlcNAc is phosphorylated to GlcNAc-6P by the PTS system, which should prevent the accumulation of intracellular GlcNAc.^64^ These results suggest three possibilities: (i) intracellular hydrolysis of COS, (ii) detection of GlcNAc-6P instead of GlcNAc, or (iii) the presence of a native non-PTS system in *E. coli*.

Although strain 3KO_A5 grown on HPLC-grade GlcNAc shows the lowest COS (Figure 4A) and specific COS (Figure 4B) production titers, it achieves the highest purity, with 83.9% of the product being the intended chitinpentaose. High-purity COS products are desirable, as different COS molecules have varying biological functions, making heterogeneous COS mixtures more challenging to utilize effectively.^65^ The COS composition was influenced by the type of GlcNAc used. With mushroom-derived GlcNAc, the COS composition shifted primarily to chitinbiose (54.2 and 52.7%) and chitintriose (23.8 and 20.5%). In contrast, HPLC-grade shrimp-derived GlcNAc resulted predominantly in chitinpentaose (83.9 and 40.0%) and chitinhexaose (16.1 and 7.2%). To ensure that the intracellular chitinbiose and chitintriose where the result of bacterial production rather than originating from the mushroom-derived GlcNAc minimal medium, a stability test was conducted. The mushroom-derived GlcNAc minimal medium was incubated under COS production (shaken for 24 h; see Section 2.2.1), confirming that the observed COS were exclusively produced by the bacterial strains (Figure S5). These findings indicate that residual components in the unpurified, mushroom-derived GlcNAc not only influence strain growth, but also alter the resulting COS composition.^66^

Despite shifts in COS composition, both strains achieved their highest COS production with mushroom-derived GlcNAc (Figure 4), underscoring the potential of this sustainable carbon source. When grown on mushroom-derived GlcNAc, strains 3KO_A5 and 4KO_A5_GlcNAc_2 produced 0.173 and 0.277 g/L COS, respectively. For strain 3KO_A5, COS production was similar between the two carbon sources when intracellular GlcNAc was excluded, being 0.103 g/L for HPLC-grade GlcNAc and 0.134 g/L for mushroom-derived GlcNAc (Table S5). Notably, strain 4KO_A5_GlcNAc_2 produced significantly more COS than strain 3KO_A5 when grown on mushroom-derived GlcNAc, with and without including intracellular GlcNAc (Table S5). In contrast, when using HPLC-grade GlcNAc, the two strains produce statistically similar amounts of combined chitinpentaose (0.0867 and 0.0706 g/L) and chitinhexaose (0.0168 and 0.0128 g/L) (Figure 4A and Table S5). These findings demonstrate the competitive potential of strain 4KO_A5_GlcNAc_2 as an alternative to strain 3KO_A5, particularly when a non-PTS GlcNAc import system is desired. This expands the possibilities for engineering efficient COS producing strains tailored to specific carbon sources.

Although using mushroom-derived GlcNAc resulted in similar or slightly higher COS production titers, it also led to a shift toward shorter-chain COS and reduced purity—both critical factors for industrial COS applications.^67,68^ Since both microbial growth and COS production are linked to UDP-GlcNAc availability, reduced growth can impact overall COS yields.^56,69,70^ The hexosamine biosynthesis pathway converts GlcNAc into UDP-GlcNAc, which serves as a precursor for both COS biosynthesis (Figure 1) and peptidoglycan production.^56,69,70^ Consequently, a metabolic trade-off exists between microbial growth and COS production.^71^ Despite the observed reduction in growth, COS production remained high when using mushroom-derived GlcNAc. This suggests that while residual components in the unpurified mushroom-derived GlcNAc negatively impact growth and COS composition, they do not necessarily inhibit COS biosynthesis. A possible explanation is that due to reduced growth, less UDP-GlcNAc is available, which has been shown to impact COS chain length.^72,73^ Due to this lower UDP-GlcNAc concentration, the synthesis of chitinbiose and chitintriose might be favored over longer chain COS.^72,73^ Addressing these limitations will be key to optimizing COS production from sustainable carbon sources.

**Figure 5:**
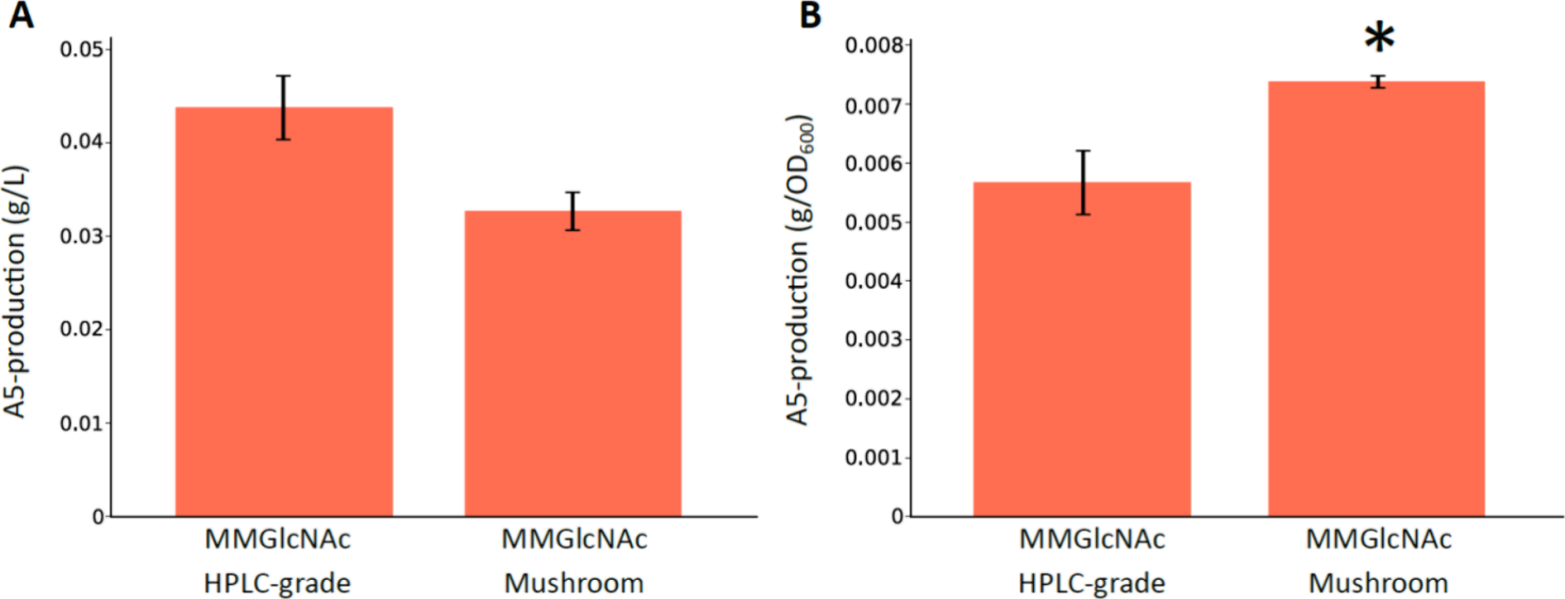
COS production of the *Escherichia coli* strain 3KO_A5 (see Table 1 for more details on the strain). A purified, mushroom-derived GlcNAc concentration of 2.8 g/L was used. Results were compared to production on commercial, HPLC-grade minimal medium. The mean over three biological replicates (*n* = 3) is shown. The error bars represent the standard error based on the mean. (A) Chitinpentaose (A5) production titers. For statistical analysis, a two-sample *t* test was performed to compare A5-production between the two carbon sources (t-stat = −2.755, *p*-value = 5.11 × 10^–02^). *0.01 < *p* < 0.05. (B) Specific chitinpentaose (A5) production titers. For statistical analysis, a two-sample *t* test was performed to compare specific A5-production between the two carbon sources (t-stat = 3.089, *p*-value = 3.66 × 10^–02^). *0.01 < *p* < 0.05. More details on the growth and A5-production titers can be found in Table S7. A5 = fully acetylated chitinpentaose, MM = Minimal medium, GlcNAc = *N*-acetylglucosamine, *Rs*NodC = NodC chitin oligosaccharide synthase from *Rhizobium* species GRH2, HPLC = high-performance liquid chromatography, COS = chitin oligosaccharides.

A case study was conducted to assess the effects of impurities in mushroom-derived GlcNAc minimal medium on COS production. To reduce impurities, the saccharification product of brown *A. bisporus* chitin was purified using preparative HPLC. This process effectively removed most contaminant COS, buffer, and Maillard reaction components, resulting in an off-white product composed of over 95% GlcNAc and a small fraction (4.1%) of what is assumed to be chitintriose (Figure 2D). The COS production (Figure 5A) and specific COS production (Figure 5B) titers of strain 3KO_A5 were reevaluated using this purified mushroom-derived GlcNAc as carbon source. Due to capacity limitations of the preparative HPLC, only a limited amount of purified GlcNAc was obtained, necessitating the use of 2.8 g/L purified GlcNAc as carbon source for strain growth. Interestingly, COS composition remained consistent across the two tested carbon sources – (i) purified, mushroom-derived GlcNAc and (ii) HPLC-grade GlcNAc – with chitinpentaose as the primary detectable component and only trace amounts of chitinhexaose, which were below the detection limit. Chitinpentaose production titers were 0.0327 and 0.0437 g/L using mushroom-derived and HPLC-grade GlcNAc, respectively. While a slight reduction in chitinpentaose production was observed with mushroom-derived GlcNAc, this difference was not statistically significant (Figure 5A). The lower chitinpentaose production titers compared to the previous COS production experiment can be attributed to the lower GlcNAc concentration used in this experiment, being 2.8 g/L compared to 13.81 g/L in the previous COS production experiment (Figure 4). Notably, specific COS production for strain 3KO_A5 was higher with both purified and nonpurified mushroom-derived GlcNAc compared to HPLC-grade GlcNAc (Figures 4B and 5B). These results suggest that the impurities responsible for the shift in COS composition were successfully removed, though future research is needed to identify the specific compound(s) involved. Importantly, purified mushroom-derived GlcNAc yielded COS compositions and production titers comparable to those obtained with HPLC-grade GlcNAc, emphasizing its potential as a sustainable and cost-effective carbon source.

Although the COS composition remained unchanged, a difference in growth was observed between the two carbon sources. Growth on purified, mushroom-derived GlcNAc resulted in an average OD_max_ of 4.43, compared to an average OD_max_ of 7.77 with HPLC-grade GlcNAc (Table S7). This growth discrepancy suggests the presence of residual components in purified mushroom-derived GlcNAc MM that hinder growth. Importantly, this indicates that the compounds responsible for the shift in COS composition are not linked to the reduced growth observed with mushroom-derived GlcNAc. To improve the usability of mushroom-derived GlcNAc, future research should focus on identifying and removing these growth-inhibiting compounds. Advanced HPLC analysis using detectors beyond the evaporative light scattering detector (ELSD) and pulsed-amperometric detector, such as UV–vis, mass spectrometry, or refractive index detectors, could improve detailed characterization of the residual components in the mushroom-derived GlcNAc mixture.

One compound likely remaining after purification is ascorbic acid, which is added to the buffer during enzymatic saccharification of mushroom-derived chitin, where it acts as a reductant for *Sm*LPMO.^74^ Ascorbic acid is known to possess antibacterial properties and can inhibit *E. coli* growth.^75^ However, it degrades significantly when heated, particularly between 85 and 95 °C for 10 min, depending on the initial concentration.^76,77^ Given that the obtained saccharification product was heated to 100 °C for 10 min, any residual ascorbic acid is likely minimal. However, the main degradation product of ascorbic acid, furfural, is toxic to the microbial metabolism and could account for the observed decrease in growth.^78,79^ To test this hypothesis, growth of the WT (Figure S6) and 3KO_A5 (Figure S7) strains was monitored on minimal medium (MM) containing HPLC-grade GlcNAc supplemented with various concentrations of ascorbic acid. The highest concentration tested corresponds to the theoretical amount that would be present if no ascorbic acid had been lost during the process, representing the initial amount added during enzymatic saccharification. The mixtures were heated to 100 °C for 10 min to replicate saccharification conditions. Growth inhibition correlated with increasing concentrations of ascorbic acid, especially in the WT strain (Figure S6). The 3KO_A5 strain also exhibited growth inhibition, albeit to a lesser extent (Figure S7). These findings suggest that ascorbic acid, or its degradation products, could contribute to the reduced growth observed with mushroom-derived GlcNAc MM. However, ascorbic acid degradation is concentration-and time-dependent.^76,77^ This means that the conditions tested may not exactly reflect the actual levels of degradation occurring during the saccharification process, which could lead to discrepancies between the experimental setup and fermentation environment. Additionally, the impact of furfural on microbial growth was only indirectly assessed. To confirm these findings, the amount of ascorbic acid and furfural present in the fermentation medium should be quantified, and ascorbic acid should be removed from the mushroom-based GlcNAc MM, for example by using activated carbons.^80^ This would further elucidate its impact on microbial growth and enhance the applicability of mushroom-derived GlcNAc as a carbon source. Additionally, further studies could include testing the growth of a wider range of microbial strains to better understand how ascorbic acid and its degradation products affect microbial communities, especially in more complex or industrial fermentation settings.

Despite these challenges, our results demonstrate that COS production remains efficient using mushroom-derived GlcNAc. The COS production titers presented in this paper are comparable to those reported in literature for microbial COS production. For instance, Mey et al. reported chitinpentaose production of 0.043 and 0.083 g/L using the 3KO_A5 *E. coli* strain grown on glucose in a 3 mL culture, with the higher yield obtained by supplementing 0.5 mM FeCl_2_ to the medium.^57^ In another study, Shi et al. used an engineered *E. coli* strain to produce 0.2071 and 0.4686 g/L COS (82.2% chitinpentaose, 13.7% chitintetraose, 1.1% chitintriose, 3.0% chitinbiose) in 100 mL shake-flask cultivation and a 5 L fed-batch bioreactor with 2.5L working volume, respectively.^81^ Similarly, Samain et al. achieved up to 2.5 g/L COS, primarily chitinpentaose, using a two-phase fed-batch cultivation strategy with an initial culture volume of 1 L and glycerol as carbon source.^72^ Ling et al. engineered *Bacillus subtilis* for COS production using both glucose and GlcNAc as carbon source.^32^ Their highest COS (85.6% chitinpentaose, 7.5% chitintetraose, 5.3% chitintriose, 1.6% chitinbiose) titer was 4.82 g/L, which was achieved in a 3 L fed-batch reactor (1.5 L working volume.^32^ In comparison, our study achieved 0.103 g/L COS (83.9% chitinpentaose and 16.1% chitinhexaose) using HPLC-grade GlcNAc in a 10 mL culture, demonstrating the potential of GlcNAc as a carbon source for microbial COS production. Additionally, when using mushroom-derived GlcNAc, we obtained COS titers of 0.134 and 0.202 g/L, consisting of chitinbiose and chitintriose. Notably, while previous studies primarily relied on larger-scale setups (100 mL–2.5 L), our system achieved high yields at just 10 mL, highlighting its efficiency and potential for further optimization and scale-up. These findings demonstrate that even at small scales, mushroom-derived GlcNAc-based COS production can achieve promising titers. Beyond titer comparisons, our study also demonstrated the ability to produce COS with high purity. With both HPLC-grade and purified mushroom-derived GlcNAc, we predominantly produced chitinpentaose, with chitinhexaose as the only additional product for the 3KO_A5 strain. This contrasts with other studies where COS mixtures contained multiple lower DP oligomers. To our knowledge, this is the first study utilizing mushroom-derived GlcNAc as a carbon source for microbial COS production, highlighting its potential for sustainable and high-purity COS biosynthesis.

Future research could explore additional strains for COS production, particularly those utilizing non-PTS GlcNAc importers, to gain deeper insights into how different import systems influence COS production. For example, assessing COS production by strain 4KO_A5_GlcNAc_1 with mushroom-derived GlcNAc MM could reveal how variations in promoter and ribosome binding site (RBS) strength for *Xc*NagP expression affect COS production titers. Given the similar growth and COS production profiles observed for strains 3KO_A5 and 4KO_A5_GlcNAc_2, testing these strains with dual carbon sources – such as mushroom-derived GlcNAc and glycerol – could provide valuable insights into decoupling growth and production.^82^ In such a scenario, glycerol would support growth, while mushroom-derived GlcNAc would serve as the substrate for COS production, potentially improving overall efficiency.

Whole genome sequencing on strains 4KO_A5_GlcNAc_1 and 4KO_A5_GlcNAc_2 could uncover genetic changes responsible for their enhanced growth and COS production compared to nonevolved counterparts. These insights could then be validated through reverse engineering. Additionally, alternative NodC genes could be explored to diversify the COS portfolio and assess whether mushroom-derived GlcNAc influences different NodC proteins in unique ways. For example, NodC proteins from *Sinorhizobium meliloti* (which predominantly produces chitintetraose) and *Sinorhizobium fredii* (which primarily yields chitinpentaose), could be studied, along with engineered NodC variants designed to modify COS composition or specificity.^56,83^ Such investigations would provide valuable insights into whether COS composition shifts depend on the NodC protein expressed and expand the range of COS products obtainable from mushroom-derived GlcNAc. This could further enhance the versatility and applicability of this sustainable carbon source for COS production.

In conclusion, this study establishes a successful proof-of-concept for producing valuable chitin oligosaccharides from chitin-rich waste streams, advancing efforts to produce valuable chemicals through renewable processes. To make this process industrially viable, future research should aim to optimize each step. First, evaluate additional mushroom strain waste streams and investigate if seasonal or batch variations could help ensure a more consistent, high-yield substrate. Furthermore, enzyme production and purification remain significant bottlenecks; efforts to extend enzyme lifespan through reusability strategies, such as immobilization, and exploring novel biocatalysts or engineered chitinases, could significantly reduce costs. The growth of the 3KO_A5 strain on mushroom-derived GlcNAc remains suboptimal compared to that on HPLC-grade GlcNAc, indicating that impurities from the saccharification process may inhibit production strain growth. Identifying these impurities and optimizing their removal will be essential for improving both growth and COS production during precision fermentation. Additionally, implementing a two-step strategy that decouples growth and production - using conventional carbon sources like glycerol for growth and mushroom-derived GlcNAc for COS production - could further enhance efficiency. Overall, this study highlights the potential of mushroom-derived GlcNAc as a sustainable carbon source for COS production, providing a foundation for scaling this renewable process to meet industrial demands and contributing to a more sustainable bioeconomy.

## Supporting Information Available

Additional tables for experimental data, analysis of variance, coding DNA sequences, spacer and ribosome binding site sequences; additional figures for chitin saccharification, *E. coli* strain growth, chitinpentaose production, and GlcNAc stability in minimal medium (PDF)

## Abbreviations

A5: fully acetylated chitinpentaose
AB: antibiotic resistance
*A. bisporus*: *Agaricus bisporus*
AcN: acetonitrile
Amp: ampicillin
CDS: coding DNA sequence
Chlo: chloramphenicol
COS: chitin oligosaccharides
CPEC: Circular Polymerase Extension Cloning
DA: degree of acetylation
DE: directed evolution
DP: degree of polymerization
*E. coli*: *Escherichia coli*
ELSD: evaporative light scattering detector
Fru: fructose
GG: Golden Gate
GlcN: glucosamine
GlcN_5_: chitopentaose
GlcN_6_: chitohexaose
GlcNAc: *N*-acetyl-d-glucosamine
GlcNAc_2_: chitinbiose
GlcNAc_5_: chitinpentaose
HILIC: hydrophilic interaction chromatography
HPAEC-PAD: high performance anion exchange chromatography–pulsed amperometric detection
HPLC: high performance liquid chromatography
IPTG: isopropyl β-d-1-thiogalactopyranoside
Kan: kanamycin
KO: knockout
LB: lysogeny broth
LPMO: lytic polysaccharide monooxygenase
MES: 2-morpholinoethanesulfonic acid
MM: minimal medium
MTP: microtiter plate
Ni-NTA: nickel-nitrilotriacetic acid
OD_600_: optical density at 600 nm
ori: origin of replication
P: phosphate
Prom: promoter
PA: pattern of acetylation
PaA5: partially acetylated chitinpentaose
paCOS: partially acetylated chitin oligosaccharides
PBS: phosphate-buffered saline
PCR: polymerase chain reaction
PMSF: phenylmethylsulfonyl fluoride
*P. ostreatus*: *Pleurotus ostreatus*
PTS: phosphotransferase system
RBS: ribosome binding site
RID: refractive index detector
*Rs*: *Rhizobium* species GRH2
*Rs*NodB: chitin oligosaccharide synthase B from *Rhizobium* species GRH2
*Rs*NodC: chitin oligosaccharide synthase C from *Rhizobium* species GRH2
SEVA: Standard European Vector Architecture
*Sm*: *Serratia marcescens*
*Sm*ChiA: Chitinase A from *Serratia marcescens*
*Sm*ChiB: Chitinase B from *Serratia marcescens*
*Sm*ChiC: Chitinase C from *Serratia marcescens*
*Sm*HEX: β-*N*-acetylhexosaminidase from *Serratia marcescens*
*Sm*LPMO: lytic polysaccharide monooxygenease 10A from *Serratia marcescens*
Term: terminator
TIR: translation initiation rate
TR: transcription rate
UTR: untranslated region
W: weight
WT: wild-type
*Xc*: *Xanthomonas campestris*
*Xc*NagP: NagP permease from *Xanthomonas campestris*.
