# Supplementary material for: Bioconversion of Mushroom Chitin-Rich Waste into Valuable Chitin Oligosaccharides Using a Combined Approach of Biocatalysis and Precision Fermentation
Source: J Agric Food Chem. 2025 Apr 14;73(16):9769–81. doi: 10.1021/acs.jafc.5c00928 (PMC12056690; doi:10.1021/acs.jafc.5c00928)
Supplement: Supplementary file 1 — jf5c00928_si_001.pdf [file jf5c00928_si_001.pdf]

Supplementary information

Bioconversion of mushroom chitin-rich waste into valuable chitin oligosaccharides using a combined approach of biocatalysis and precision fermentation

Alex Windels[1†], Luna Declerck[1†], Sofie Snoeck[1], Wouter Demeester[1], Chiara Guidi[1], Tom Desmet[1], Marjan De Mey[1*]

[1]Centre for Synthetic Biology, Ghent University, Ghent, Belgium

[†]These authors contributed equally to this work.

* Correspondence:

Marjan De Mey

marjan.demey@ugent.be

1 Supplementary tables

Table S1: List of DNA sequences (coding DNA sequences (CDSs), promoter (P), 5' untranslated region (UTR) and terminator sequences) used in this study. Start and stop codons are indicated with lower-case letters. *Rs* = *Rhizobium* species GRH2, *Sm* = *Serratia marcescens*, *Xc* = *Xanthomonas campestris*, UTR = untranslated region.

Gene	Coding sequence
*Sm*ChiA	5'-<br>atgAAATATCTGCTGCCGACCGCAGAAGCAGGTCTGCTGCTGCTGTTAGCAGCACCGCAGATTGCA<br>GCCGCACCGGGTAAACCGACCATTGCATGGGGTAATACCAAATTTGCAATTGTGGAAGTTGATC<br>AGGCAGCAACCGCATATAACAATCTGGTGAAAGTTAAAAATGCAGCCGATGTTAGCGTTAGCTG<br>GAATCTGTGGAATGGTGATGCAGGCACCACCGCCAAAATTCTGCTGAATGGTAAAGAAGCATGG<br>TCAGGTCCGAGCACCGGTAGCAGCGGCACCGCAAATTTCAAAGTTAATAAAGGTGGTCGCTACC<br>AGATGCAGGTTGCACTGTGTAATGCAGATGGTTGTACCGCAAGTGATGCAACCGAAATTGTTGTT<br>GCAGATACCGATGGTAGCCATCTGGCACCGCTGAAAGAACCGCTGCTGGAAAAAAACAAACCGT<br>ATAAACAGAATAGCGGTAAAGTGGTGGGTAGCTATTTTGTTGAATGGGGTGTTTATGGTCGCAAC<br>TTTACCGTTGATAAAATTCCGGCACAGAATCTGACCCATCTGCTGTATGGTTTTATTCCGATTTGT<br>GGTGGCAATGGCATTAACGATAGCCTGAAAGAAATTGAAGGTAGCTTTCAGGCACTGCAGCGTA<br>GCTGTCAGGGTCGTGAAGATTTTAAAGTTAGCATCCATGATCCGTTTGCAGCACTGCAGAAAGCA<br>CAGAAAGGTGTTACCGCATGGGATGATCCGTATAAAGGTAATTTTGGTCAGCTGATGGCACTGA<br>AACAGGCACATCCGGATCTGAAAATCCTGCCGAGCATTGGTGGTTGGACCCTGAGCGATCCGTTT<br>TTTTTCATGGGTGATAAAGTGAAACGTGATCGTTTTGTTGGTAGCGTGAAAGAATTTCTGCAGAC<br>CTGGAAATTTTTCGATGGCGTTGATATCGATTGGGAATTTCCTGGTGGTAAAGGTGCAAATCCGA<br>ATCTGGGTAGTCCGCAGGATGGTGAAACCTATGTTCTGCTGATGAAAGAACTGCGTGCAATGCTG<br>GATCAGCTGAGCGCAGAAACCGGTCGTAAATATGAACTGACCAGCGCAATTAGCGCAGGCAAAG<br>ATAAAATTGATAAGGTGGCATATAATGTGGCCCAGAATAGCATGGATCATATCTTTCTGATGAGC<br>TACGATTTCTATGGTCCGTTTGACCTGAAAAATCTGGGTCATCAGACCGCACTGAATGCACCGGC<br>ATGGAAACCGGATACCGCCTATACCACCGTTAATGGTGTTAATGCACTGCTGGCACAGGGTGTTA<br>AACCGGGAAAAGTTGTTGTTGGCACCGCCATGTATGGTCGCGGTTGGACCGGTGTGAATGGTTAT<br>CAGAATAACATTCCGTTTACCGGTACAGCAACCGGTCCGGTTAAAGGCACCTGGAAAAATGGTA<br>TTGTTGATTATCGTCAGATCGCAGGTCAGTTTATGAGCGGTGAATGGCAGTATACCTATGATGCA<br>ACAGCCGAAGCACCGTATGTTTTTAAACCGAGTACCGGTGATCTGATTACCTTTGATGATGCACG<br>TAGCGTTCAGGCCAAAGGTAAATATGTTCTGGATAAACAGTTAGGTGGTCTGTTTAGCTGGGAAA<br>TTGATGCAGATAATGGCGATATTCTGAACAGCATGAATGCAAGCCTGGGTAATAGTGCCGGTGTT<br>CAAGGTAGTGGCCTCGAGCACCACCACCACCACCACtga-3'
*Sm*ChiB	5'-<br>atgAGCACCCGTAAAGCCGTTATTGGCTATTATTTCATTCCGACCAACCAGATCAACAACTATACC<br>GAAACCGATACCAGCGTTGTTCCGTTTCCGGTTAGCAATATTACACCGGCAAAAGCAAAACAGCT<br>GACCCATATTAACTTTAGCTTTCTGGATATCAACAGCAATCTGGAATGTGCATGGGATCCTGCAA<br>CCAATGATGCAAAAGCACGTGATGTTGTTAATCGTCTGACCGCACTGAAAGCACATAATCCGAG<br>CCTGCGTATTATGTTTAGCATTGGTGGTTGGTATTACAGCAATGATCTGGGTGTTAGCCATGCCA<br>ATTATGTTAATGCAGTTAAAACACCGGCAGCGCGTACCAAATTTGCACAGAGCTGTGTTCGTATC<br>ATGAAAGATTATGGTTTTGATGGCGTGGATATCGATTGGGAATATCCGCAGGCAGCCGAAGTTG<br>ATGGTTTTATTGCAGCACTGCAAGAAATTCGTACCCTGCTGAACCAGCAGACCATTGCCGATGGT<br>CGTCAGGCACTGCCGTATCAGCTGACAATTGCCGGTGCCGGTGGTGCATTTTTTCTGAGTCGTTAT<br>TATAGCAAACTGGCCCAGATTGTTGCACCGCTGGATTATATCAATCTGATGACCTATGATCTGGC<br>AGGTCCGTGGGAGAAAATTACCAATCATCAGGCAGCACTGTTTGGTGATGCAGCAGGTCCGACC<br>TTTTATAACGCACTGCGTGAAGCAAATTTAGGTTGGAGCTGGGAAGAACTGACCCGTGCATTTCC<br>GAGTCCGTTTAGCCTGACCGTTGATGCAGCCGTTCAGCAGCATCTGATGATGGAAGGTGTTCCGA<br>GCGCAAAAATTGTTATGGGTGTGCCGTTTTATGGTCGTGCATTTAAAGGTGTTAGCGGTGGTAAT<br>GGTGGTCAGTATAGCAGCCATAGCACACCGGGTGAAGATCCGTATCCGAATGCAGATTATTGGC<br>TGGTTGGCTGTGATGAATGTGTTCGTGATAAAGATCCGCGTATTGCAAGCTATCGTCAGCTGGAA<br>CAAATGCTGCAGGGTAATTATGGTTATCAGCGTCTGTGGAACGATAAAACCAAAACACCGTATCT<br>GTATCATGCACAGAATGGTCTGTTTGTGACGTATGATGATGCCGAAAGCTTTAAATACAAGGCCA<br>AATACATTAAACAGCAGCAGTTAGGTGGTGTGATGTTTTGGCATCTGGGTCAAGATAATCGTAAT<br>GGTGATCTGCTGGCAGCCCTGGATCGCTATTTTAACGCAGCAGATTATGATGATAGCCAGCTGGA<br>TATGGGCACCGGTCTGCGTTATACCGGTGTTGGTCCGGGTAATCTGCCGATTATGACCGCACCAG<br>CCTATGTTCCGGGTACAACCTATGCACAGGGTGCACTGGTTAGCTATCAGGGTTATGTTTGGCAG

	ACCAAATGGGGTTATATTACCAGCGCACCGGGTAGCGATAGCGCATGGCTGAAAGTTGGTCGCC<br>TGGCAGGTAGTGGCCTCGAGCACCACCACCACCACCACtga-3’
*Sm*ChiC	5’-<br>atgAGCACCAACAATACCATTAATGCAGTTGCAGCAGATGATGCAGCAATTATGCCGAGCATTGCA<br>AACAAAAAAATCCTGATGGGCTTTTGGCATAACTGGGCAGCCGGTGCAAGTGATGGTTATCAGC<br>AGGGTCAGTTTGCAAATATGAATCTGACCGATATTCCGACCGAATATAACGTTGTTGCAGTGGCA<br>TTTATGAAAGGTCAGGGCATTCCGACCTTTAAACCGTATAATCTGAGCGATACCGAATTTCGTCG<br>TCAGGTTGGTGTTCTGAATAGCCAGGGTCGTGCAGTTCTGATTAGCTTAGGTGGTGCAGATGCAC<br>ATATTGAACTGAAAACCGGTGATGAGGATAAACTGAAAGATGAAATTATTCGCCTGGTGGAAGT<br>GTATGGTTTTGATGGTCTGGATATTGATCTGGAACAGGCAGCAATTGGTGCAGCAAATAACAAA<br>ACCGTTCTGCCTGCAGCACTGAAAAAAGTGAAAGATCATTATGCAGCCCAGGGCAAAAACTTTA<br>TTATCAGCATGGCACCGGAATTTCCGTATCTGCGTACCAATGGCACCTATCTGGATTATATCAAT<br>GCACTGGAAGGCTATTATGATTTTATTGCACCGCAGTATTATAACCAAGGTGGTGATGGTATTTG<br>GGTCGATGAACTGAATGCATGGATTACCCAGAATAACGATGCCATGAAAGAGGACTTTCTGTATT<br>ATCTGACAGAAAGCCTGGTTACCGGTACACGTGGTTATGCAAAAATTCCGGCAGCCAAATTTGTT<br>ATTGGTCTGCCGAGCAATAATGATGCCGCAGCAACCGGTTATGTTGTTAATAAACAGGCAGTGTA<br>TAACGCCTTTAGCCGTCTGGATGCAAAAAACCTGAGCATTAAAGGTCTGATGACCTGGTCTATTA<br>ATTGGGATAATGGTAAAAGCAAAGCCGGTGTTGCATATAACTGGGAATTCAAAACCCGTTATGC<br>ACCGCTGATTCAAGGTGGCGTTACACCGCCTCCGGGTAAACCGAATGCACCGACCGCACTGACC<br>GTTGCAGAACTGGGTGCAACCAGCCTGAAACTGAGCTGGGCAGCAGCCACCGGTGCATTTCCGA<br>TTGCAAGCTATACCGTTTATCGTAATGGTAATCCGATTGGTCAGACCGCAGGTCTGTCACTGGCA<br>GATGGTGGTCTGACACCGGCAACACAGTATAGCTATTTCGTTACCGCAACCGATAGTCAGGGTAA<br>TACCAGCCTGCCGAGTAGCGCACTGGCAGTTAAAACCGCAAATGATGGCACCCCTCCGGATCCT<br>GGTGCTCCGGAATGGCAGAATAATCATTCATATAAAGCGGGTGATGTGGTGAGCTATAAAGGCA<br>AAAAATACACCTGTATTCAGGCCCATACCAGCAATGCAGGTTGGACACCGGATGCAGCATTTAC<br>CCTGTGGCAGCTGATTGCAGGTAGTGGCCTCGAGCACCACCACCACCACCACtga-3’
*Sm*LPMO10A	5’-<br>atgAACAAAACCAGTCGTACCCTGCTGAGCCTGGGTCTGCTGAGCGCAGCAATGTTTGGTGTTAGC<br>CAGCAGGCAAATGCACATGGTTATGTTGAAAGTCCGGCAAGCCGTGCATATCAGTGTAAACTGC<br>AGCTGAATACCCAGTGTGGTAGCGTTCAGTATGAACCGCAGAGCGTTGAAGGTCTGAAAGGTTTT<br>CCGCAGGCAGGTCCGGCAGATGGTCATATTGCAAGCGCAGATAAAAGCACCTTTTTTGAACTGG<br>ATCAGCAGACACCGACACGTTGGAATAAACTGAATCTGAAAACCGGTCCGAATAGCTTTACCTG<br>GAAACTGACCGCACGTCATAGCACCACCAGTTGGCGCTATTTTATCACCAAACCGAATTGGGATG<br>CAAGCCAGCCGCTGACACGTGCATCATTTGATCTGACCCCGTTTTGTCAGTTTAATGATGGTGGT<br>GCAATTCCGGCAGCACAGGTTACCCATCAGTGCAATATTCCAGCAGATCGTAGCGGTAGCCATGT<br>TATTCTGGCAGTTTGGGATATTGCAGATACCGCAAATGCCTTTTATCAGGCCATTGATGTTAATCT<br>GAGCAAGCTCGAGCACCACCACCACCACCACtga-3’
*Sm*HEX	5’-<br>atgGATCAGCAGCTGGTTGATCAGCTGTCTCAGCTGAAACTGAATGTTAAAATGCTGGATAATCGC<br>GCAGGCGAAAATGGTGTTGATTGTGCAGCACTGGGTGCAGATTGGGCAAGCTGTAATCGTGTTCT<br>GTTTACCCTGAGCAATGATGGTCAGGCAATTGATGGTAAAGATTGGGTGATCTATTTTCATAGTC<br>CGCGTCAGACCCTGCGTGTTGATAATGATCAGTTTAAAATCGCACATCTGACCGGTGATCTGTAT<br>AAACTGGAACCGACCGCAAAATTTTCAGGTTTTCCGGCAGGTAAAGCAGTTGAAATTCCGGTTGT<br>TGCAGAATATTGGCAGCTGTTTCGTAATGATTTTCTGCCTCGTTGGTATGCAACCAGCGGTGATG<br>CAAAACCGAAAATGTTAGCAAATACCGATACCGAAAACCTGGATCAGTTTGTTGCACCGTTTACA<br>GGTGATCAGTGGAAACGTACCAAAGACGATAAAAACATTCTGATGACACCGGCAAGCCGTTTTG<br>TTAGCAATGCCGATCTGCAGACACTGCCTGCCGGTGCACTGCGTGGTAAAATTGTTCCGACACCG<br>ATGCAGGTTAAAGTTCATGCACAGGATGCGGATCTGCGTAAAGGTGTTGCACTGGATCTGAGCA<br>CCCTGGTTAAACCGGCAGCAGATGTTGTTAGCCAGCGTTTTGCACTGCTGGGTGTTCCGGTTCAG<br>ACCAATGGTTATCCGATTAAAACCGATATTCAGCCTGGTAAATTCAAAGGTGCAATGGCAGTTAG<br>CGGTGCCTATGAACTGAAAATTGGTAAGAAAGAAGCCCAGGTTATTGGCTTTGATCAGGCAGGC<br>GTGTTTTATGGTCTGCAGAGCATTCTGAGCCTGGTTCCGAGTGATGGTAGCGGCAAAATTGCAAC<br>CCTGGATGCAAGTGATGCACCGCGTTTTCCGTATCGTGGTATTTTTCTGGATGTTGCCCGTAACTT<br>TCATAAAAAGGATGCAGTTCTGCGTCTGCTGGATCAGATGGCAGCATATAAACTGAACAAATTTC<br>ACTTTCACCTGAGCGACGATGAAGGTTGGCGTATTGAGATTCCGGGTCTGCCGGAACTGACCGAA<br>GTTGGTGGTCAGCGTTGTCATGATCTGAGCGAAACCACATGCCTGCTGCCGCAGTATGGTCAGGG<br>TCCTGATGTTTATGGTGGTTTCTTTAGCCGTCAGGATTATATCGACATCATCAAATATGCACAGGC<br>ACGTCAGATTGAAGTGATTCCGGAAATTGATATGCCTGCACATGCACGTGCCGCAGTTGTTAGCA<br>TGGAAGCACGTTATAAGAAACTGCATGCCGCAGGCAAAGAACAAGAAGCAAACGAATTTCGTCT

	GGTGGATCCGACCGATACCAGCAATACCACCAGTGTTCAGTTCTTTAATCGTCAGAGCTATCTGA<br>ATCCGTGTCTGGATAGCAGCCAGCGCTTTGTTGATAAAGTTATTGGTGAAATTGCCCAGATGCAT<br>AAAGAAGCAGGTCAGCCGATCAAAACCTGGCATTTTGGTGGTGATGAAGCCAAAAACATCCGTT<br>TAGGTGCAGGTTATACCGATAAAGCAAAACCGGAACCTGGTAAAGGTATTATTGATCAGAGCAA<br>CGAAGATAAACCGTGGGCGAAAAGCCAGGTGTGTCAGACCATGATCAAAGAAGGTAAAGTTGCC<br>GATATGGAACATCTGCCGAGCTATTTTGGTCAAGAAGTTAGCAAACTGGTTAAAGCCCATGGTAT<br>TGATCGTATGCAGGCATGGCAGGATGGTCTGAAAGATGCAGAAAGCAGCAAAGCATTTGCGACC<br>AGCCGTGTTGGTGTGAATTTTTGGGATACCCTGTATTGGGGTGGTTTTGATAGCGTTAATGATTGG<br>GCCAATAAAGGCTATGAAGTTGTTGTGAGCAACCCGGATTATGTGTATATGGATTTTCCGTACGA<br>AGTGAACCCGGATGAACGTGGTTATTATTGGGGCACCCGTTTTAGTGATGAACGTAAAGTTTTTA<br>GCTTCGCACCGGATAATATGCCGCAGAATGCCGAAACCAGCGTTGATCGTGATGGTAATCATTTT<br>AATGCCAAAAGCGATAAACCTTGGCCTGGTGCGTATGGTCTGAGCGCACAACTGTGGTCAGAAA<br>CCCAGCGTACCGATCCGCAGATGGAATATATGATTTTTCCGCGTGCACTGAGCGTTGCAGAACGT<br>AGCTGGCATCGTGCAGGTTGGGAACAAGATTATCGTGCCGGTCGTGAATATAAAGGTGGTGAAA<br>CCCATTTTGTGGATACACAGGCACTGGAAAAAGATTGGCTGCGTTTTGCGAATATTCTGGGTCAG<br>CGTGAACTGGCAAAACTGGATAAAGGCGGTGTTGCATATCGTCTGCCGGTTCCGGGTGCACGTGT<br>TGCAGCGGGAAAACTGGAAGCAAATATTGCACTGCCTGGTCTGGGTATTGAATATAGCACCGAT<br>GGTGGTAAACAGTGGCAGCGTTATGATGCCAAAGCCAAACCTGCAGTGAGCGGTGAAGTTCAGG<br>TTCGTAGCGTTAGTCCGGATGGTAAACGTTATAGCCGTGCAGAAAAAGTGCTCGAGCACCACCA<br>CCACCACCACtga-3’
*Xcnag*P	5’-<br>atgACTACGGCGCGTCCGGCAAACCCGGTTGTTTCCATTGCGATTGTTGGCGTTCTGTTTTTCATTA<br>TCGGTTTCTTCACCTGGATCAACGGCCCGCTGATTACTTTCGTCCGTCTGGCGTTTGACCTGAATG<br>AAGTTAACGCCTTCCTGGTGCTGATGGTCTTTTACCTGTCTTACTTTTTTCTGGCCCTGCCGTCCTC<br>TTGGATTCTGAAACGTACCGGCATGAAAAAAGGTCTGGCACTGTCTCTGGTTGTAATGGCTGTAG<br>GTGCAGCAGGTTTCGGCCAGTTCGCGACTCAACGTTGGTACCCAGGTGCGCTGGGTGGTCTGTTT<br>GTCATCGGCTCCGGTCTGGCTCTGCTGCAGACCGCCATCAACCCGTATATCTCCATCCTGGGCCC<br>GATCGAATCTGCAGCACGCCGTATCGCACTGATGGGTATTTGTAACAAAATCGCCGGTATTCTGG<br>CTCCGATTCTGATCGGCTCCCTGGTTCTGCATGGCATTGGCGACCTGTCTGCACAGGTTGCGTCCG<br>CCGACGCTACCACTAAAGAAACCCTGCTGACTGCGTTCGCAGCGAAGATCCACGCGCCGTATCTG<br>GTTATGTCCGGTGTTCTGCTGCTGCTGGCAATCGGTGTACTGTTCAGCCCGCTGCCGGAACTGAA<br>GCCGAGCGAAGCCAACGCGACCCCTGGCGCAACCGGCGGTGTGCAGAAATCTTCTATTTTCCAGT<br>TCCCGCACCTGTGGCTGGGTGTTCTGTGTCTGTTCGTTTACGTAGGTGTTGAAGTGATGGCCGGCG<br>ATGCGATCGGCACCTACGGCCATGGCTTTAACCTGCCGCTGGACAGCACTAAGATTTTCACTTCC<br>TATACCCTGGGCGCGATGCTGCTGGGCTACATCGCGGGTCTGGTTCTGATCCCGCGTGTAATCTC<br>TCAAGCGCGCTACCTGTCTGTGAGCGCTGTTCTGGGTGTTCTGTTCTCTCTGGGCGCGCTGTTTAC<br>TCACGGTTATGTGAGCGTAGGCTTCGTTGCAGCACTGGGCTTTGCTAATGCGATGATGTGGCCGG<br>CTATCTTTCCGCTGGCGATTCGTGGCCTGGGTCGTTTCACCGAGATCGGTTCTGCCCTGCTGGTAA<br>TGGGCATTGCTGGCGGTGCGATCATTCCGCAGCTGTTTGCAATCCTGAAACAGCACTATGACTTC<br>CAGGTGGTTTTTGCTGCCCTGATGGTTCCGTGTTACCTGTACATCCTGTTTTACTCTCTGCGCGGC<br>CATCGTGTTGGCCTGCCTGCTCAGGCCAAAtaa-3’
*Rsnod*C	5’-<br>atgGACCTGCTGAACACGATTGGTATTGGTGCTGTCTCCTGCTACGCTCTGCTGTCAACGGCTCATA<br>AGTCGATGCAAACCCTGTATGCCCAGCCGAAAGATCAAAGCTCTGCATCAGAAGATTTTGCTTTC<br>CTGCCGTCGGTGGATATTATCGTTCCGTGTTATAACGAAAATCCGCATACCTTTAGCGAATGCCT<br>GGCGTCTATTGCCAACCAGGATTATGCGGGCAAACTGCGTGTGTACGTGGTTGATGACGGTAGTG<br>CCAATCGTGAAAAGCTGGAACGCGTTCATCACACCTACGCAGGCGATCCGCGTTTTGACTTCATC<br>CTGCTGCGTGAAAACGTGGGTAAGCGTAAGGCACAGATTGCAGCAATCCGTGGCAGTTCCGGTG<br>ATCTGGTGCTGAATGTTGATAGCGACTCTACCCTGGCATCAGACGTCGTGACGAAACTGGCTCTG<br>AAGATGCAGAACCCGGAAATTGGCGCAGCTATGGGTCAACTGACCGCGTCTAACCGTAATGATA<br>CCTGGCTGACGCGCCTGATCGACATGGAATATTGGCTGGCCTGTAATGAAGAACGTGCAGCACA<br>GGCACGTTTTGGTGCAGTGATGTGCTGTTGCGGTCCGTGCGCAATGTATCGTCGCTCAGCTCTGCT<br>GTCGCTGCTGGATCAGTACGAAAGCCAATTTTTCCGTGGCAAACCGTCTGATTTTGGTGAAGATC<br>GCCATCTGACCATTCTGATGCTGAAGGCGGGCTTCCGT ACG GAT TAT GTT CCG GAC GCC ATC<br>GCA GCT ACC GTT GTC CCG GAT CGT ATG GGT CCG TAC CTG CGC CAG<br>CAACTGCGTTGGGCACGCAGCACCTTCCGTGATACGCTGCTGGCTCTGCGTCTGCTGCCGGGTCT<br>GGATCACTATATTACGCTGGACGTTATCGGTCAGAACCTGGGTCCGCTGCTGCTGGCACTGGCTG<br>TCCTGACCGGTGTACTGCAAGTGGCACTGACCGCTACGGTCCCGCTGTGGACCGTGATGATGATT<br>GCATCAATGACGATGATCCGTTGTGCAGTTGCAGCAGTCCGTGCACGTCAGCTGCGCTTTCTGGT<br>TTTCTCGCTGCATACCCCGATTAACCTGTTTTTCCTGCTGCCGATGAAAGCGTACGCCCTGTGCAC<br>GCTGAGTAACTCCGATTGGCTGAGTCGCTCATCGCCGGCGAATAAAACCTCCGCCGGCGGTGAA

	CACCCGACCACGGAAGCAAGTGCTGGCGGTACCTCCGGCAACGCGACGCCGCTGCGTCGCCTGA ACCTGGCTCGTGACTCCTCTACCGTTACCCCGGCTGGTGTCTACTCCGATGATtga-3’
nonCDS	5’- AAATGAACGATTTCTTAGTCGGCGTTATAGTAAGTCACTCTTTTTCAGCGGTATTTTAAAGATGA GAAAGCGATGGTCAAGCGTGGTCTGCCTGAAGTCT-3’
**Promoter**	**DNA sequence**
P_{nagP2}	5’- CTGAAGTCTCCAAGCAGCTCCAGCCTACAATTGGCGGAAGGCCGTCAAGGCCGCATCGGTGATG AATTTCTTGACAGTTATTAGGGACAATACTATAATGTGTGG -3’
P_{nagP3}	5’- CTGAAGTCTCCAAGCAGCTCCAGCCTACAATTGGCGGAAGGCCGTCAAGGCCGCATCGGTGATG AATTTCTTGACACCCGTCCGTCCGATTGGTATAATGTGTGG -3’
P_{14}	5’- AACAGATAAAACGAAAGGCCCAGTCTTTCGACTGAGCCTTTCGTTTTATTTGATGCCTTAATTAA AAGGGGTCTCGACTCAGGAACTCTTCATTCTATAAGTTTCTTGACATCTTGGCCGGCATATGGTAT AATAGGGCCCC-3’
**5’UTR**	**DNA sequence**
P_{nagP2} 5’UTR	5’- ATCCCCTTTGTTTAACTTTAAGAAGCCACCAGCTATAAGAAGGATGT -3’
P_{nagP3} 5’UTR	5’-ATCCCCTTTGTTTAACTTTAAGAAGCCACCAGCTATAAGACGGATGT-3’
T7 5’UTR	5’-CTCTAGAAATAAGTTTTGTTTAACTTTAAGAAGGAGATATACATA-3’
**Terminator**	**DNA sequence**
TT5-T7term	5’- ACAACCCTCAAGAGAAAATGTAATCACACTGGCTCACCTTCGGGTGGGCCTTTCTGCGTTTATAA GGAGACACTTTATGTTTAAGAAG-3’
T7 terminator	5’-CTAGCATAACCCCTTGGGGCCTCTAAACGGGTCTTGAGGGGTTTTTTG-3’
TT3-rrnD1-T1	5’- GGGAACTGCCAGGCATCAAATAAAACGAAAGGCTCAGTCGAAAGACTGGGCCTTTCGTTTTATC TGTTGTTTGTCGG TGAACGCTCTCCTG-3’
TT7-M13	5’- AAAGCAAGCTGATAAACCGATACAATTAAAGGCTCCTTTTGGAGCCTTTTTTTTTGGAGATTTTC AACATGAAAAAATTATTATT-3’

Table S2: Backbones of the plasmids used in precision fermentation experiments. The sequence of interest are inserted at **NNNNNNNNNN** for backbones P[BR322][Kan] and P[BR322][Amp]. Plasmids containing

backbone P[15A][Chl] were constructed using an in-house assembly platform (See section 2.1 for more information). Transcription unit 1 is indicated as **NNNNNNNNNN** and transcription unit 2 as *NNNNNNNNNN*. The sequences of the regulatory elements and DNA sequences inserted are listed in Table S1. Kan = kanamycin, Amp = ampicilin, Chlo = chloramphenicol.

**Backbone**	**Coding sequence**
P[BR322]<br>[Kan]	5’-<br>AGTCCTAGGATGCTAGCTATGTGGGCTTACATGGCGATAGCTAGACTGGGCGGTTTTATGGACAGCA<br>AGCGAACCGGAATTGCCAGCTGGGGCGCCCTCTGGTAAGGTTGGGAAGCCCTGCAAAGTAAACTGG<br>ATGGCTTTCTTGCCGCCAAGGATCTGATGGCGCAGGGGATCAAGATCTGATCAAGAGACAGGATGA<br>GGATCGTTTCGCATGATTGAACAAGATGGATTGCACGCAGGTTCTCCGGCCGCTTGGGTGGAGAGGC<br>TATTCGGCTATGACTGGGCACAACAGACAATCGGCTGCTCTGATGCCGCCGTGTTCCGGCTGTCAGC<br>GCAGGGGCGCCCGGTTCTTTTTGTCAAGACCGACCTGTCCGGTGCCCTGAATGAACTGCAGGACGAG<br>GCAGCGCGGCTATCGTGGCTGGCCACGACGGGCGTTCCTTGCGCAGCTGTGCTCGACGTTGTCACTG<br>AAGCGGGAAGGGACTGGCTGCTATTGGGCGAAGTGCCGGGGCAGGATCTCCTGTCATCTCACCTTGC<br>TCCTGCCGAGAAAGTATCCATCATGGCTGATGCAATGCGGCGGCTGCATACGCTTGATCCGGCTACC<br>TGCCCATTCGACCACCAAGCGAAACATCGCATCGAGCGAGCACGTACTCGGATGGAAGCCGGTCTTG<br>TCGATCAGGATGATCTGGACGAAGAGCATCAGGGGCTCGCGCCAGCCGAACTGTTCGCCAGGCTCA<br>AGGCGCGCATGCCCGACGGCGAGGATCTCGTCGTGACCCATGGCGATGCCTGCTTGCCGAATATCAT<br>GGTGGAAAATGGCCGCTTTTCTGGATTCATCGACTGTGGCCGGCTGGGTGTGGCGGACCGCTATCAG<br>GACATAGCGTTGGCTACCCGTGATATTGCTGAAGAGCTTGGCGGCGAATGGGCTGACCGCTTCCTCG<br>TGCTTTACGGTATCGCCGCTCCCGATTCGCAGCGCATCGCCTTCTATCGCCTTCTTGACGAGTTCTTCT<br>GAGCGGGACTCTGGGGTTCGAAATGACCGACCAAGCGACGCCCAACCTGCCATCACGAGATTTCGA<br>TTCCACCGCCGCCTTCCCCCCATGAACAGAAATCCCCCTTACACGGAGGCATCAGTGACCAAACAGG<br>AAAAAACCGCCCTTAACATGGCCCGCTTTATCAGAAGCCAGACATTAACGCTTCTGGAGAAACTCAA<br>CGAGCTGGACGCGGATGAACAGGCAGACATCTGTGAATCGCTTCACGACCACGCTGATGAGCTTTAC<br>CGCAGCTGCCTCGCGCGTTTCGGTGATGACGGTGAAAACCTCTGACACATGCAGCTCCCGGAGACGG<br>TCACAGCTTGTCTGTAAGCGGATGCCGGGAGCAGACAAGCCCGTCAGGGCGCGTCAGCGGGTGTTG<br>GCGGGTGTCGGGGCGCAGCCATGACCCAGTCACGTAGCGATAGCGGAGTGTATACTGGCTTAACTAT<br>GCGGCATCAGAGCAGATTGTACTGAGAGTGCACCATATGCGGTGTGAAATACCGCACAGATGCGTA<br>AGGAGAAAATACCGCATCAGGCGCTCTTCCGCTTCCTCGCTCACTGACTCGCTGCGCTCGGTCGTTCG<br>GCTGCGGCGAGCGGTATCAGCTCACTCAAAGGCGGTAATACGGTTATCCACAGAATCAGGGGATAA<br>CGCAGGAAAGAACATGTGAGCAAAAGGCCAGCAAAAGGCCAGGAACCGTAAAAAGGCCGCGTTGC<br>TGGCGTTTTTCCATAGGCTCCGCCCCCCTGACGAGCATCACAAAAATCGACGCTCAAGTCAGAGGTG<br>GCGAAACCCGACAGGACTATAAAGATACCAGGCGTTTCCCCCTGGAAGCTCCCTCGTGCGCTCTCCT<br>GTTCCGACCCTGCCGCTTACCGGATACCTGTCCGCCTTTCTCCCTTCGGGAAGCGTGGCGCTTTCTCA<br>TAGCTCACGCTGTAGGTATCTCAGTTCGGTGTAGGTCGTTCGCTCCAAGCTGGGCTGTGTGCACGAA<br>CCCCCCGTTCAGCCCGACCGCTGCGCCTTATCCGGTAACTATCGTCTTGAGTCCAACCCGGTAAGAC<br>ACGACTTATCGCCACTGGCAGCAGCCACTGGTAACAGGATTAGCAGAGCGAGGTATGTAGGCGGTG<br>CTACAGAGTTCTTGAAGTGGTGGCCTAACTACGGCTACACTAGAAGGACAGTATTTGGTATCTGCGC<br>TCTGCTGAAGCCAGTTACCTTCGGAAAAAGAGTTGGTAGCTCTTGATCCGGCAAACAAACCACCGCT<br>GGTAGCGGTGGTTTTTTTGTTTGCAAGCAGCAGATTACGCGCAGAAAAAAAGGATCTCAAGAAGATC<br>CTTTGATCTTTTCTACGGGGTCTGACGCTCAGTGGAACGAAAACTCACGTTAAGGGATTTTGGTCATG<br>AGATTATCAAAAAGGATCTTCACCTAGATCCTTTTAAATTAAAAATGAAGTTTTAAATCAATCTAAA<br>GTATATATGAGTAAACTTGGTCTGACAGAGCTGGCACGACAGGTTTCCCGACTGGAAATAGACGTCG<br>CCTCAGCTTGTGACGAAAAGTGCCACCTGACGTCATTAAT**NNNNNNNNN**-3’
P[BR322]<br>[Amp]	5’-<br>CTGAAAGGAGGAACTATATCCGGATTGGCGAATGGGACGCGCCCTGTAGCGGCGCATTAAGCGCGG<br>CGGGTGTGGTGGTTACGCGCAGCGTGACCGCTACACTTGCCAGCGCCCTAGCGCCCGCTCCTTTCGC<br>TTTCTTCCCTTCCTTTCTCGCCACGTTCGCCGGCTTTCCCCGTCAAGCTCTAAATCGGGGGCTCCCTTT<br>AGGGTTCCGATTTAGTGCTTTACGGCACCTCGACCCCAAAAAACTTGATTAGGGTGATGGTTCACGT<br>AGTGGGCCATCGCCCTGATAGACGGTTTTTCGCCCTTTGACGTTGGAGTCCACGTTCTTTAATAGTGG<br>ACTCTTGTTCCAAACTGGAACAACACTCAACCCTATCTCGGTCTATTCTTTTGATTTATAAGGGATTT<br>TGCCGATTTCGGCCTATTGGTTAAAAAATGAGCTGATTTAACAAAAATTTAACGCGAATTTTAACAA<br>AATATTAACGTTTACAATTTCAGGTGGCACTTTTCGGGGAAATGTGCGCGGAACCCCTATTTGTTTAT<br>TTTTCTAAATACATTCAAATATGTATCCGCTCATGAGACAATAACCCTGATAAATGCTTCAATAATAT<br>TGAAAAAGGAAGAGTATGAGTATTCAACATTTCCGTGTCGCCCTTATTCCCTTTTTTGCGGCATTTTG<br>CCTTCCTGTTTTTGCTCACCCAGAAACGCTGGTGAAAGTAAAAGATGCTGAAGATCAGTTGGGTGCA<br>CGAGTGGGTTACATCGAACTGGATCTCAACAGCGGTAAGATCCTTGAGAGTTTTCGCCCCGAAGAAC

	GTTTTCCAATGATGAGCACTTTTAAAGTTCTGCTATGTGGCGCGGTATTATCCCGTATTGACGCCGGG<br>CAAGAGCAACTCGGTCGCCGCATACACTATTCTCAGAATGACTTGGTTGAGTACTCACCAGTCACAG<br>AAAAGCATCTTACGGATGGCATGACAGTAAGAGAATTATGCAGTGCTGCCATAACCATGAGTGATA<br>ACACTGCGGCCAACTTACTTCTGACAACGATCGGAGGACCGAAGGAGCTAACCGCTTTTTTGCACAA<br>CATGGGGGATCATGTAACTCGCCTTGATCGTTGGGAACCGGAGCTGAATGAAGCCATACCAAACGA<br>CGAGCGTGACACCACGATGCCTGCAGCAATGGCAACAACGTTGCGCAAACTATTAACTGGCGAACT<br>ACTTACTCTAGCTTCCCGGCAACAATTAATAGACTGGATGGAGGCGGATAAAGTTGCAGGACCACTT<br>CTGCGCTCGGCCCTTCCGGCTGGCTGGTTTATTGCTGATAAATCTGGAGCCGGTGAGCGTGGGTCTCG<br>CGGTATCATTGCAGCACTGGGGCCAGATGGTAAGCCCTCCCGTATCGTAGTTATCTACACGACGGGG<br>AGTCAGGCAACTATGGATGAACGAAATAGACAGATCGCTGAGATAGGTGCCTCACTGATTAAGCAT<br>TGGTAACTGTCAGACCAAGTTTACTCATATATACTTTAGATTGATTTAAAACTTCATTTTTAATTTAA<br>AAGGATCTAGGTGAAGATCCTTTTTGATAATCTCATGACCAAAATCCCTTAACGTGAGTTTTCGTTCC<br>ACTGAGCGTCAGACCCCGTAGAAAAGATCAAAGGATCTTCTTGAGATCCTTTTTTTCTGCGCGTAATC<br>TGCTGCTTGCAAACAAAAAAACCACCGCTACCAGCGGTGGTTTGTTTGCCGGATCAAGAGCTACCAA<br>CTCTTTTTCCGAAGGTAACTGGCTTCAGCAGAGCGCAGATACCAAATACTGTCCTTCTAGTGTAGCCG<br>TAGTTAGGCCACCACTTCAAGAACTCTGTAGCACCGCCTACATACCTCGCTCTGCTAATCCTGTTACC<br>AGTGGCTGCTGCCAGTGGCGATAAGTCGTGTCTTACCGGGTTGGACTCAAGACGATAGTTACCGGAT<br>AAGGCGCAGCGGTCGGGCTGAACGGGGGGTTCGTGCACACAGCCCAGCTTGGAGCGAACGACCTAC<br>ACCGAACTGAGATACCTACAGCGTGAGCTATGAGAAAGCGCCACGCTTCCCGAAGGGAGAAAGGCG<br>GACAGGTATCCGGTAAGCGGCAGGGTCGGAACAGGAGAGCGCACGAGGGAGCTTCCAGGGGGAAA<br>CGCCTGGTATCTTTATAGTCCTGTCGGGTTTCGCCACCTCTGACTTGAGCGTCGATTTTTGTGATGCTC<br>GTCAGGGGGGCGGAGCCTATGGAAAAACGCCAGCAACGCGGCCTTTTTACGGTTCCTGGCCTTTTGC<br>TGGCCTTTTGCTCACATGTTCTTTCCTGCGTTATCCCCTGATTCTGTGGATAACCGTATTACCGCCTTT<br>GAGTGAGCTGATACCGCTCGCCGCAGCCGAACGACCGAGCGCAGCGAGTCAGTGAGCGAGGAAGCG<br>GAAGAGCGCCTGATGCGGTATTTTCTCCTTACGCATCTGTGCGGTATTTCACACCGCATATATGGTGC<br>ACTCTCAGTACAATCTGCTCTGATGCCGCATAGTTAAGCCAGTATACACTCCGCTATCGCTACGTGAC<br>TGGGTCATGGCTGCGCCCCGACACCCGCCAACACCCGCTGACGCGCCCTGACGGGCTTGTCTGCTCC<br>CGGCATCCGCTTACAGACAAGCTGTGACCGTCTCCGGGAGCTGCATGTGTCAGAGGTTTTCACCGTC<br>ATCACCGAAACGCGCGAGGCAGCTGCGGTAAAGCTCATCAGCGTGGTCGTGAAGCGATTCACAGAT<br>GTCTGCCTGTTCATCCGCGTCCAGCTCGTTGAGTTTCTCCAGAAGCGTTAATGTCTGGCTTCTGATAA<br>AGCGGGCCATGTTAAGGGCGGTTTTTTCCTGTTTGGTCACTGATGCCTCCGTGTAAGGGGGATTTCTG<br>TTCATGGGGGTAATGATACCGATGAAACGAGAGAGGATGCTCACGATACGGGTTACTGATGATGAA<br>CATGCCCGGTTACTGGAACGTTGTGAGGGTAAACAACTGGCGGTATGGATGCGGCGGGACCAGAGA<br>AAAATCACTCAGGGTCAATGCCAGCGCTTCGTTAATACAGATGTAGGTGTTCCACAGGGTAGCCAGC<br>AGCATCCTGCGATGCAGATCCGGAACATAATGGTGCAGGGCGCTGACTTCCGCGTTTCCAGACTTTA<br>CGAAACACGGAAACCGAAGACCATTCATGTTGTTGCTCAGGTCGCAGACGTTTTGCAGCAGCAGTCG<br>CTTCACGTTCGCTCGCGTATCGGTGATTCATTCTGCTAACCAGTAAGGCAACCCCGCCAGCCTAGCCG<br>GGTCCTCAACGACAGGAGCACGATCATGCGCACCCGTGGGGCCGCCATGCCGGCGATAATGGCCTG<br>CTTCTCGCCGAAACGTTTGGTGGCGGGACCAGTGACGAAGGCTTGAGCGAGGGCGTGCAAGATTCC<br>GAATACCGCAAGCGACAGGCCGATCATCGTCGCGCTCCAGCGAAAGCGGTCCTCGCCGAAAATGAC<br>CCAGAGCGCTGCCGGCACCTGTCCTACGAGTTGCATGATAAAGAAGACAGTCATAAGTGCGGCGAC<br>GATAGTCATGCCCCGCGCCCACCGGAAGGAGCTGACTGGGTTGAAGGCTCTCAAGGGCATCGGTCG<br>AGATCCCGGTGCCTAATGAGTGAGCTAACTTACATTAATTGCGTTGCGCTCCGTTATCCCCTGATTCT<br>GTGGATAACCGTATTACCGCCTTTGAGTGAGCTGATACCGCTCGCCGCAGCCGAACGACCGAGCGCA<br>GCGAGTCAGTGAGCGAGGAAGCGGAAGAGCGCCTGATGCGGTATTTTCTCCTTACGCATCTGTGCGG<br>TATTTCACACCGCATATGGTGCACTCTCAGTACAATCTGCTCTGATGCCGCATAGTTAAGCCAGTATA<br>CACTCCGCTATCGCTACGTGACTGGGTCATGGCTGCGCCCCGACACCCGCCAACACCCGCTGACGCG<br>CCCTGACGGGCTTGTCTGCTCCCGGCATCCGCTTACAGACAAGCTGTGACCGTCTCCGGGAGAGCTC<br>GATATCCCGGGCGGCCGC**NNNNNNNNN**-3’
P[15A]<br><br>[Chl]	5’-<br>ATCATAAGCGAGACCGAGTGATGCAACTGTGCGGATGTGAAGGTTGTCTTCGCAGTAAGTACGCCTT<br>CTCCGAAGACTTGAGTACTAGTCTTGGACTCCTGTTGATAGATCCAGTAATGACCTCAGAACTCCATC<br>TGGATTTGTTCAGAACGCTCGGTTGCCGCCGGGCGTTTTTTATTGGTGAGAATCCAGGGGTCCCCAAT<br>AATTACGATTTAAATTGGCGAAAATGACACGTTGATCGGCACGTAAGAGGTTCCAACTTTCACCATA<br>ATGAAATAAGATCACTACCGGGCGTATTTTTTGAGTTATCGAGATTTTCAGGAGCTAAGGAAGCTAA<br>AATGGAGAAAAAAATCACTGGATATACCACCGTTGATATATCCCAATGGCATCGTAAAGAACATTTT<br>GAGGCATTTCAGTCAGTTGCTCAATGTACCTATAACCAGACCGTTCAGCTGGATATTACGGCCTTTTT<br>AAAGACCGTAAAGAAAAATAAGCACAAGTTTTATCCGGCCTTTATTCACATTCTTGCCCGCCTGATG<br>AATGCTCATCCGGAATTTCGTATGGCAATGAAAGACGGTGAGCTGGTGATATGGGATAGTGTTCACC<br>CTTGTTACACCGTTTTCCATGAGCAAACTGAAACGTTTTCATCGCTCTGGAGTGAATACCACGACGAT<br>TTCCGGCAGTTTCTACACATATATTCGCAAGATGTGGCGTGTTACGGTGAAAACCTGGCCTATTTCCC<br>TAAAGGGTTTATTGAGAATATGTTTTTCGTATCAGCCAATCCCTGGGTGAGTTTCACCAGTTTTGATT<br>TAAACGTGGCCAATATGGACAACTTCTTCGCCCCCGTTTTCACCATGGGCAAATATTATACGCAAGG

CGACAAGGTGCTGATGCCGCTGGCGATTCAGGTTCATCATGCCGTTTGTGATGGCTTCCATGTCGGC
AGAATGCTTAATGAATTACAACAGTACTGCGATGAGTGGCAGGGCGGGGCGTAATTTGACNNNNGT
CCTTTTCCGCTGCATAACCCTGCTTCGGGGTCATTATAGCGATTTTTTCGGTATATCCATCCTTTTTCG
CACGATATACAGGATTTTGCCAAAGGGTTCGTGTAGACTTTCCTTGGTGTATCCAACGGCGTCAGCC
GGGCAGGATAGGTGAAGTAGGCCCACCCGCGAGCGGGTGTTCCTTCTTCACTGTCCCTTATTCGCAC
CTGGCGGTGCTCAACGGGAATCCTGCTCTGCGAGGCTGGCCGTAGGCCGGCCCTAGAAATATTTTAT
CTGATTAATAAGATGATCTTCTTGAGATCGTTTTGGTCTGCGCGTAATCTCTTGCTCTGAAAACGAAA
AAACCGCCTTGCAGGGCGGTTTTTCGAAGGTTCTCTGAGCTACCAACTCTTTGAACCGAGGTAACTG
GCTTGGAGGAGCGCAGTCACCAAAACTTGTCCTTTCAGTTTAGCCTTAACCGGCGCATGACTTCAAG
ACTAACTCCTCTAAATCAATTACCAGTGGCTGCTGCCAGTGGTGCTTTTGCATGTCTTTCCGGGTTGG
ACTCAAGACGATAGTTACCGGATAAGGCGCAGCGGTCGGACTGAACGGGGGGTTCGTGCATACAGT
CCAGCTTGGAGCGAACTGCCTACCCGGAACTGAGTGTCAGGCGTGGAATGAGACAAACGCGGCCAT
AACAGCGGAATGACACCGGTAAACCGAAAGGCAGGAACAGGAGAGCGCACGAGGGAGCCGCCAGG
GGGAAACGCCTGGTATCTTTATAGTCCTGTCGGGTTTCGCCACCACTGATTTGAGCGTCAGATTTCGT
GATGCTTGTCAGGGGGGCGGAGCCTATGGAAAAACGGCTTTTGCCGCGGCCCTCTCACTTCCCTGTT
AAGTATCTTCCTGGCATCTTCCAGGAAATCTCCGCCCCGTTCGTAAGCCATTTCCGCTCGCCGCAGTC
GAACGACCGAGCGTAGCGAGTCAGTGAGCGAGGAAGCGGAATATATCCGGCGCGCCCAGCTGTCTA
GGGCGGCGGATTTGTCCTACTCAGGAGAGCGTTCACCGACAAACAACAGATAAAACGAAAGGCCCA
GTCTTTCGACTGAGCCTTTCGTTTTATTTGATGCCTTAATTAAAAGGGGTCTCGACTCAGGAACT**NNN**
NNNNNNNGCGAAGTGACACGAATTACTTATCTGGCAGGAGTATCGTCCGTAGTTTCAACTGTTCTGT
GCATACGGCCCTGAAAGACTATTGATTACGAATATAAGGAAAACCACTAAATGAACGATTTCTTAGT
CGGCGTTATAGTAAGTCACTCTTTTTCAGCGGTATTTTAAAGATGAGAAAGCGATGGTCAAGCGTGG
TCTGCCTGAAGTCTACTC*NNNNNNNNNN*CAAGTTTGTACGGCAAGATGCGA-3’

Table S3: Spacer and ribosome binding site sequences used for *Xc*NagP production. The transcription rate and translation initiation rate of the promoter and RBS sequence were calculated using the Salis calculator.[37,38] The spacer region was fully randomized, the RBS was semi-randomized (MHGVMGGATS; M=A/C, H=A/C/T, V=A/C/G, S=C/G). The underlined nucleotides were not varied. RBS = ribosome binding site. TR = transcription rate, TIR = translation initiation rate, au = arbitrary unit.

**Variant**	**Spacer**	**RBS**	**TR (au)**	**TIR (au)**
*nag*P1	GTTATTAGGGACAATAC	AAGAAGGATGT	27037.10	902.61
*nag*P2	CCCGTCCGTCCGATTGG	AAGACGGATGT	30567.93	28.30

Table S4: 24h saccharification yield and product profile for the three sources of chitin tested in this study. The yields are expressed as percentages of total weight (% w/w) and include detailed fractions of GlcNAc and its oligomers (GlcNAc2 to GlcNAc6). All reactions were performed in triplicate. GlcNAc = *N*-acetylglucosamine, COS = chitin oligosaccharides. *A. bisporus* = *Agaricus bisporus*, *P. ostreatus* = *Pleurotus ostreatus.*

**Chitin source**	**COS yield (% w/w)**	**GlcNAc fraction (% w/w)**	**GlcNAc$_2$ fraction (% w/w)**	**GlcNAc$_3$ fraction (% w/w)**	**GlcNAc$_4$ fraction (% w/w)**	**GlcNAc$_5$ fraction (% w/w)**	**GlcNAc$_6$ fraction (% w/w)**
A. bisporus white	28 ± 3	75 ± 8	1.1 ± 0.2	14 ± 4	3.0 ± 0.4	4.8 ± 0.5	2.2 ± 0.3

A. bisporus brown	40 ± 2	77 ± 3	0.60 ± 0.06	7.2 ± 0.4	4.5 ± 0.4	6.6 ± 0.7	3.8 ± 0.3
P. ostreatus	6.1 ± 0.8	55 ± 8	1.8 ± 0.3	18 ± 4	5 ± 1	11 ± 4	10 ± 1

Table S5: Statistical tests for COS production of strain 3KO_A5 and strain 4KO_A5_GlcNAc_2 on mushroom-derived GlcNAc and HPLC-grade GlcNAc. One-way ANOVA and Tukey HSD to correct for pairwise comparison were conducted on COS production (g/L) and specific production titers (g/OD_{600}) of all COS and COS with a degree of polymerization (DP) > 1. Significant values ($p < 0.05$) are indicated in bold. More details on the strains and plasmids are given in Tables 1, S1, S2 and S3. KO = knockout, A5 = fully acetylated chitinpentaose, GlcNAc = *N*-acetylglucosamine, COS = chitin oligosaccharides, DP = degree of polymerization, OD_{600} = optical density at 600 nm, prod = production, HPLC = high performance liquid chromatography.

**Strain (GlcNAc-source)**	**Strain (GlcNAc-source)**	**COS prod (g/L)**	**Specific COS prod (g/OD_{600})**	**COS (DP > 1) prod (g/L)**	**Specific COS (DP > 1) prod (g/OD_{600})**
**ANOVA**	P-value	**3.07E-07**	**3.31E-05**	**6.97E-05**	**4.63E-03**
	F-value	213.05	54.03	43.17	11.18
3KO_A5 (HPLC-grade)	3KO_A5 (mushroom)	**1.00E-04**	**1.75E-02**	8.29E-02	**3.29E-02**
3KO_A5 (HPLC-grade)	4KO_A5_GlcNAc_2 (HPLC-grade)	**0.00E+00**	**0.00E+00**	3.97E-01	**9.10E-03**
3KO_A5 (HPLC-grade)	4KO_A5_GlcNAc_2 (mushroom)	**0.00E+00**	**2.20E-03**	**2.00E-04**	**6.00E-03**
3KO_A5 (mushroom)	4KO_A5_GlcNAc_2 (HPLC-grade)	5.94E-02	**2.00E-04**	**1.46E-02**	5.06E-01
3KO_A5 (mushroom)	4KO_A5_GlcNAc_2 (mushroom)	**0.00E+00**	3.07E-01	**1.60E-03**	5.12E-01
4KO_A5_GlcNAc_2 (HPLC-grade)	4KO_A5_GlcNAc_2 (mushroom)	**1.00E-4**	**8.00E-04**	**1.00E-04**	9.98E-01

Table S6: COS production results of *Escherichia coli* strains 3KO_A5 and 4KO_A5_GlcNAc_2 when grown on minimal medium supplemented with 13.81 g/L HPLC-grade or mushroom-derived GlcNAc. More details

on the strains and plasmids are given in Tables 1, S1, S2 and S3. KO = knockout , A = *N*-acetylglucosamine (A from acetylated), HPLC = high performance liquid chromatography, GlcNAc = *N*-acetylglucosamine, OD_{600} = optical density at 600 nm, COS = chitin oligosaccharides.

**Strain**	**Replicate**	**A1 (g/L)**	**A2 (g/L)**	**A3 (g/L)**	**A5 (g/L)**	**A6 (g/L)**	**OD_{600}**
**HPLC-grade GlcNAc**							
3KO_A5	1	0	0	0	0.0859	0.0158	7.6
3KO_A5	2	0	0	0	0.0859	0.0172	7.8
3KO_A5	3	0	0	0	0.0883	0.0172	8.2
4KO_A5_GlcNAc_2	2	0.101	0	0	0.0694	0.0131	3.5
4KO_A5_GlcNAc_2	3	0.127	0	0	0.0718	0.0124	4.6
**Mushroom-derived GlcNAc**							
3KO_A5	1	0.0424	0.0940	0.0435	0	0	7.8
3KO_A5	2	0.0406	0.0854	0.0385	0	0	6.9
3KO_A5	3	0.0316	0.101	0.0410	0	0	7.1
4KO_A5_GlcNAc_2	1	0.0634	0.149	0.0659	0	0	9.9
4KO_A5_GlcNAc_2	2	0.0999	0.136	0.0410	0	0	8.2
4KO_A5_GlcNAc_2	3	0.0622	0.149	0.0659	0	0	11.5

Table S7: COS production and OD_{600} measurement at 24h of *Escherichia coli* strain 3KO_A5 when grown on minimal medium containing purified, mushroom-derived GlcNAc or commercial, HPLC-grade, shrimp-derived GlcNAc (= HPLC-grade GlcNAc). More details on the strain and plasmid are given in Tables 1, S1, S2 and S3. Three replicates per carbon source were used. OD_{600} = optical density measured at 600 nm, GlcNAc = *N*-acetylglucosamine, HPLC = high performance liquid chromatography, A5 = fully acetylated chitinpentaose.

**Replicate**	**Carbon source**	**A5 (g/L)**	**OD_{600}**
1	Mushroom-derived GlcNAc	0.03376	4.7

2	Mushroom-derived GlcNAc	0.02870	3.8
3	Mushroom-derived GlcNAc	0.03554	4.8
1	HPLC-grade GlcNAc	0.04663	8.7
2	HPLC-grade GlcNAc	0.03685	7.5
3	HPLC-grade GlcNAc	0.04769	7.1

2. Supplementary Figures

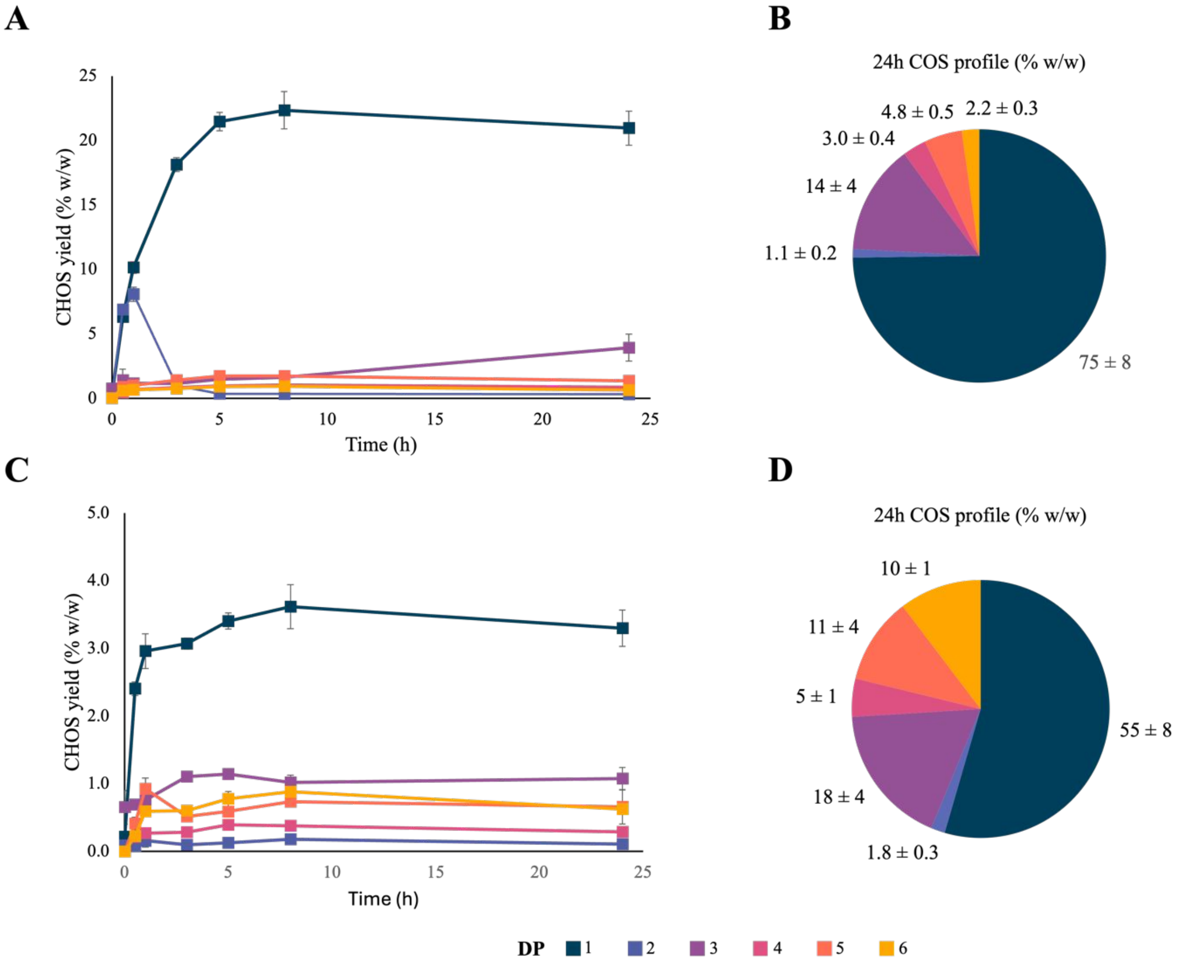

Figure S1: Saccharification of chitin derived from *Agaricus bisporus* and *Pleurotus ostreatus* waste. A. COS production during the biocatalytic saccharification process for *A. bisporus* white chitin. The mean over three biological replicates (n = 3) is shown, with error bars depicting standard error of the mean. B. *A. bisporus* white COS product profile after 24h enzymatic reaction. C. COS production during the biocatalytic saccharification process for *P. ostreatus* chitin. D. *P. ostreatus* COS product profile after 24h enzymatic reaction. Colors depict the analyzed chitin oligosaccharide, dark blue, GlcNAc; light blue, chitinbiose; purple, chitintriose; pink, chitintetraose; orange, chitinpentaose; yellow, chitinhexaose. *A. bisporus* = *Agaricus bisporus*, *P. ostreatus* = *Pleurotus ostreatus*, COS = chitin oligomers, GlcNAc = *N*-acetylglucosamine.

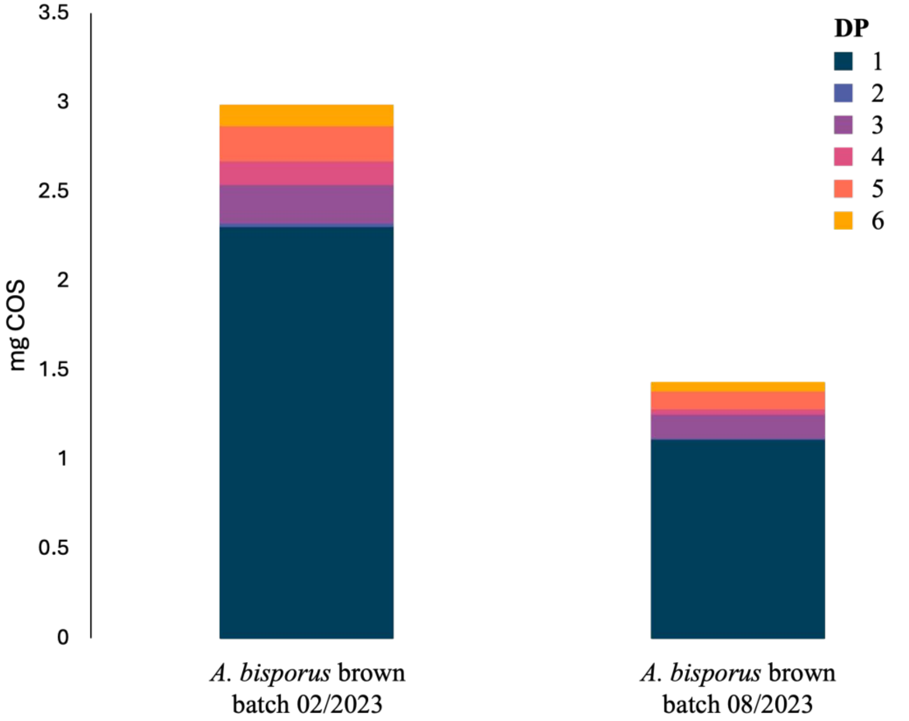

Figure S2: Batch differences in the biocatalytic conversion of *Agaricus bisporus* brown chitin. Chitin extraction was performed on stipes of *A. bisporus* brown mushrooms harvested in February 2023 and in August 2023 (Inagro, Belgium). 15 mg/mL chitin was combined with 15 mg enzyme mixture and incubated on 42 °C for 24h. COS were measured with HPAEC-PAD. Colors depict the analyzed chitin oligosaccharide, dark blue, GlcNAc; light blue, chitinbiose; purple, chitintriose; pink, chitintetraose; orange, chitinpentaose; yellow, chitinhexaose. COS = chitin oligosaccharides, DP = degree of polymerization, GlcNAc = *N*-acetylglucosamine, *A. bisporus* = *Agaricus bisporus*, HPAEC-PAD = high performance anion exchange chromatography –pulsed amperometric detection.

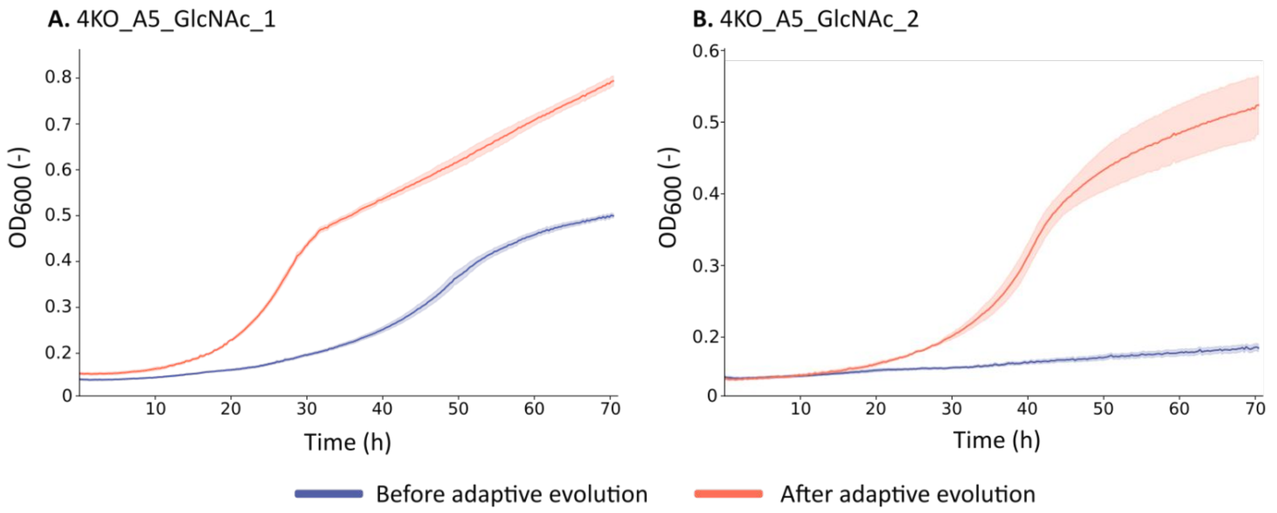

Figure S3**:** Growth experiment of *Escherichia coli* strains 4KO_A5_GlcNAc_1 and 4KO_A5_GlcNAc_2 (see Table 1 for more details on the strains) on HPLC-grade GlcNAc minimal medium to test the difference in growth before and after performing adaptive evolution. The lines represent the OD_{600} mean, surrounded by the standard error on the mean over four replicates. **A.** Growth curves of the evolved strain 4KO_A5_GlcNAc_1 and its non-evolved counterpart. **B.** Growth curves of the evolved strain 4KO_A5_GlcNAc_2 and its non-

evolved counterpart. GlcNAc = *N*-acetylglucosamine, OD_{600} = optical density at 600 nm, KO = knockout, A5 = fully acetylated chitinpentaose, HPLC = high performance liquid chromatography.

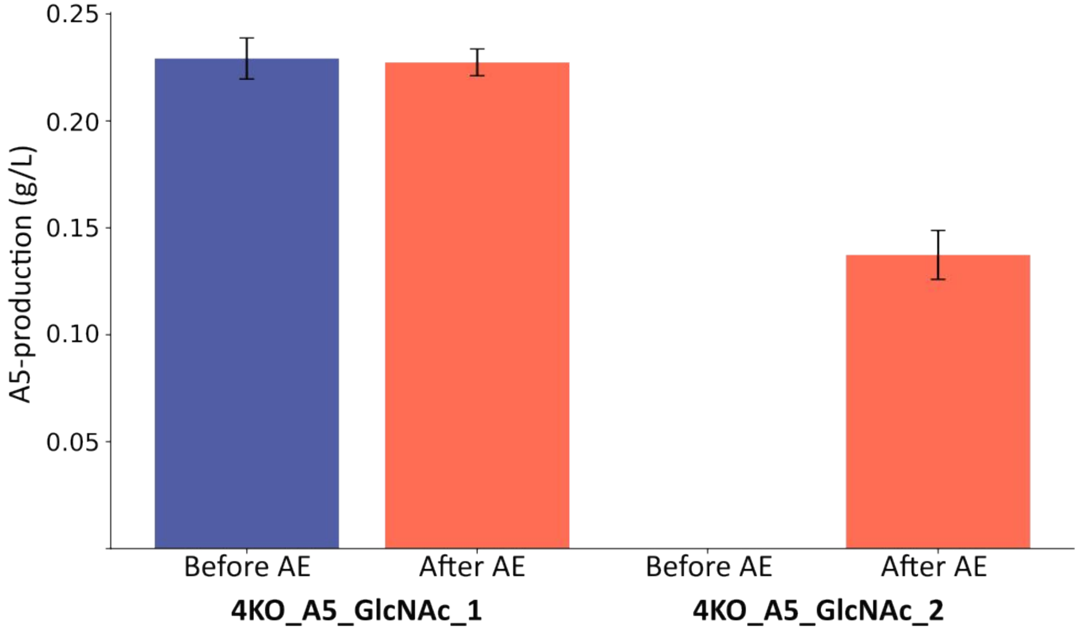

Figure S4: Chitinpentaose production (A5) of *Escherichia coli* strains 4KO_A5_GlcNAc_1 and 4KO_A5_GlcNAc_2 (see Table 1 for more details on the strains) on HPLC-grade GlcNAc minimal medium to test the difference in chitinpentaose production before and after performing adaptive evolution. Bars represent the mean values and error bars the standard error on the mean of chitinpentaose production based on three biological replicates. GlcNAc = *N*-acetylglucosamine, OD_{600} = optical density at 600 nm, KO = knockout, A5 = fully acetylated chitinpentaose, AE = adaptive evolution, HPLC = high performance liquid chromatography.

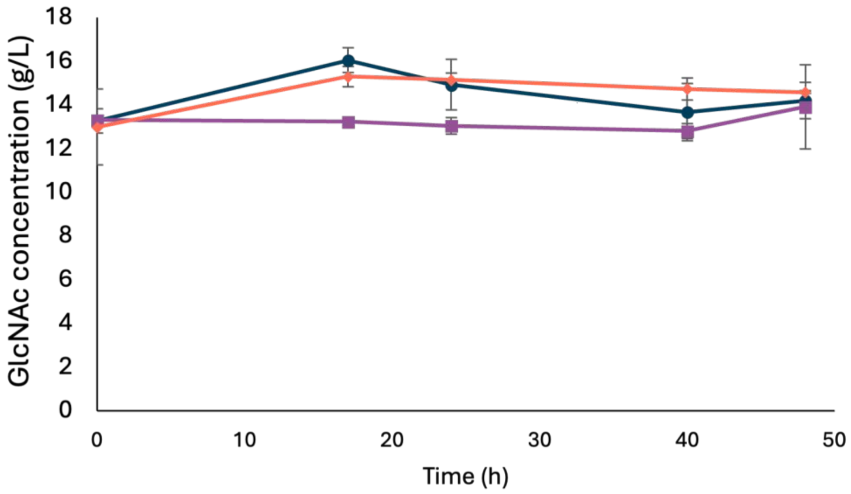

Figure S5: GlcNAc stability in minimal medium. 13.8 mg/mL GlcNAc was dissolved in filter sterilized minimal medium and incubated at 30 °C on 350 rpm for 48h. GlcNAc concentrations were measured using HPAEC-PAD. The mean over three biological replicates (n = 3) is shown, with error bars depicting standard error of the mean. Colors depict the GlcNAc fraction, unpurified GlcNAc after enzymatic hydrolysis of *A. bisporus* brown derived chitin; purple, prep-LC purified GlcNAc after enzymatic hydrolysis of *A. bisporus* brown derived chitin; dark blue, commercial GlcNAc (Merck); orange. GlcNAc = *N*-acetylglucosamine, *A. bisporus* = *Agaricus*

bisporus, HPAEC-PAD = high performance anion exchange chromatograpy – pulsed amperometric detection. Prep-LC = preparative liquid chromatography.

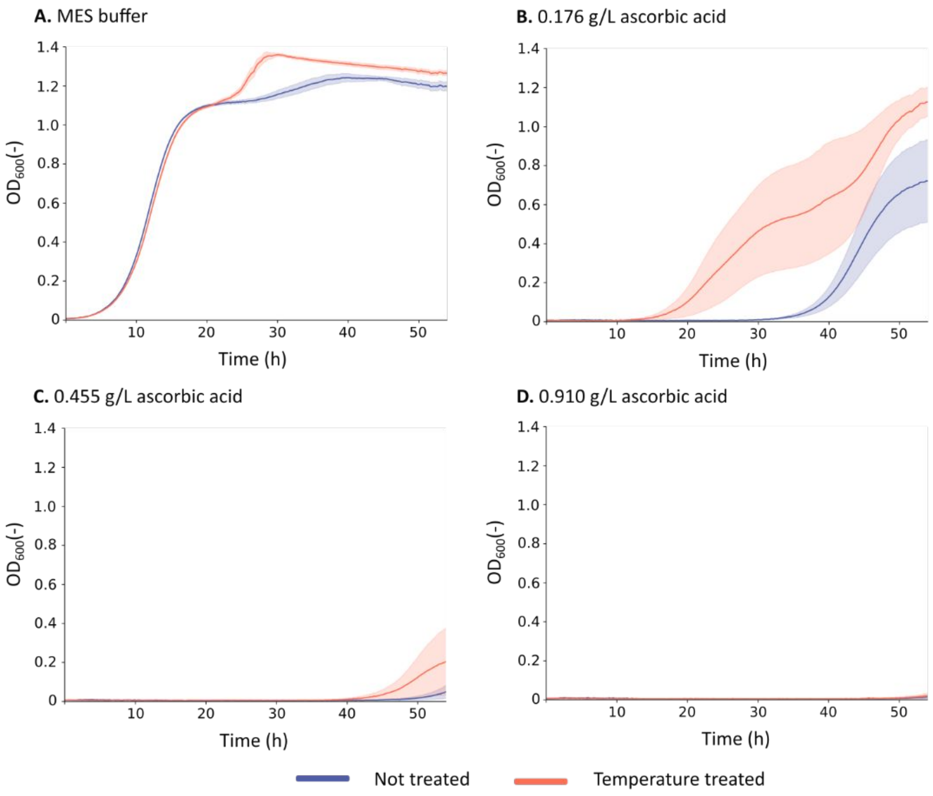

Figure S6: Growth experiment of *Escherichia coli* wild-type (WT) strain on HPLC-grade GlcNAc minimal medium (MM) to test the effect of ascorbic acid on growth. For the temperature treated curves, the HPLC-grade GlcNAc and the ascorbic acid or MES buffer were heated at 100°C for 10 minutes before being added to the MM. The lines represent the OD_{600} mean, surrounded by the standard error on the mean over four replicates. **A.** Growth of WT strain with 0.61 mg MES added, either non-treated or temperature treated. **B.** Growth of WT strain with 0.176 g/L ascorbic acid added, either non-treated or temperature treated. **C.** Growth of WT strain with 0.455 g/L ascorbic acid added, either non-treated or temperature treated. **D.** Growth of WT strain with 0.910 g/L ascorbic acid added, either non-treated or temperature treated. OD_{600} = optical density at 600 nm. MES = 2-morpholinoethanesulfonic acid, HPLC = high performance liquid chromatography.

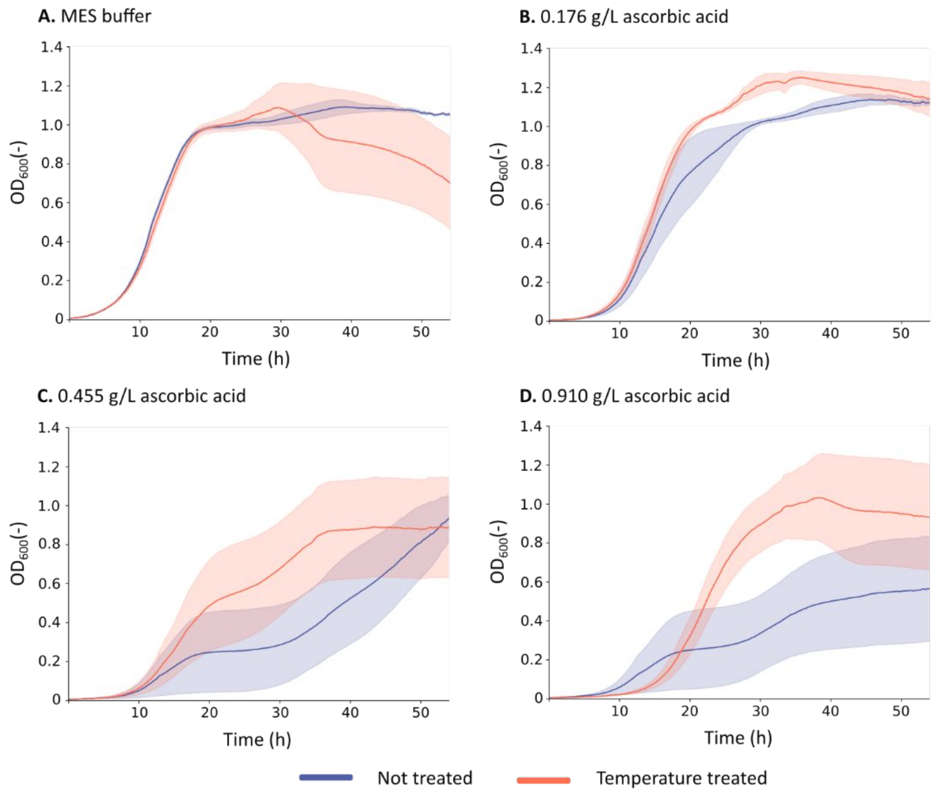

Figure S7: Growth experiment of *Escherichia coli* 3KO_A5 strain (see Table 1 for more details on the strain) on HPLC-grade GlcNAc minimal medium (MM) to test the effect of ascorbic acid on growth. For the temperature treated curves, the HPLC-grade GlcNAc and the ascorbic acid or MES buffer were heated at 100°C for 10 minutes before being added to the MM. The lines represent the OD_{600} mean, surrounded by the standard error on the mean over four replicates. **A.** Growth of 3KO_A5 strain with 0.61 mg MES added, either non-treated or temperature treated. **B.** Growth of 3KO_A5 strain with 0.176 g/L ascorbic acid added, either non-treated or temperature treated. **C.** Growth of 3KO_A5 strain with 0.455 g/L ascorbic acid added, either non-treated or temperature treated. **D.** Growth of 3KO_A5 strain with 0.910 g/L ascorbic acid added, either non-treated or temperature treated. OD_{600} = optical density at 600 nm. MES = 2-morpholinoethanesulfonic acid, HPLC = high performance liquid chromatography.
